# Switchgrass Genomic Diversity, Ploidy, and Evolution: Novel Insights from a Network-Based SNP Discovery Protocol

**DOI:** 10.1371/journal.pgen.1003215

**Published:** 2013-01-17

**Authors:** Fei Lu, Alexander E. Lipka, Jeff Glaubitz, Rob Elshire, Jerome H. Cherney, Michael D. Casler, Edward S. Buckler, Denise E. Costich

**Affiliations:** 1 Institute for Genomic Diversity, Cornell University, Ithaca, New York, United States of America; 2 Agricultural Research Service, United States Department of Agriculture, Ithaca, New York, United States of America; 3 Department of Crop and Soil Sciences, Cornell University, Ithaca, New York, United States of America; 4 Agricultural Research Service, United States Department of Agriculture, Madison, Wisconsin, United States of America; 5 Department of Agronomy, University of Wisconsin–Madison, Madison, Wisconsin, United States of America; The University of North Carolina at Chapel Hill, United States of America

## Abstract

Switchgrass (*Panicum virgatum* L.) is a perennial grass that has been designated as an herbaceous model biofuel crop for the United States of America. To facilitate accelerated breeding programs of switchgrass, we developed both an association panel and linkage populations for genome-wide association study (GWAS) and genomic selection (GS). All of the 840 individuals were then genotyped using genotyping by sequencing (GBS), generating 350 GB of sequence in total. As a highly heterozygous polyploid (tetraploid and octoploid) species lacking a reference genome, switchgrass is highly intractable with earlier methodologies of single nucleotide polymorphism (SNP) discovery. To access the genetic diversity of species like switchgrass, we developed a SNP discovery pipeline based on a network approach called the Universal Network-Enabled Analysis Kit (UNEAK). Complexities that hinder single nucleotide polymorphism discovery, such as repeats, paralogs, and sequencing errors, are easily resolved with UNEAK. Here, 1.2 million putative SNPs were discovered in a diverse collection of primarily upland, northern-adapted switchgrass populations. Further analysis of this data set revealed the fundamentally diploid nature of tetraploid switchgrass. Taking advantage of the high conservation of genome structure between switchgrass and foxtail millet (*Setaria italica* (L.) P. Beauv.), two parent-specific, synteny-based, ultra high-density linkage maps containing a total of 88,217 SNPs were constructed. Also, our results showed clear patterns of isolation-by-distance and isolation-by-ploidy in natural populations of switchgrass. Phylogenetic analysis supported a general south-to-north migration path of switchgrass. In addition, this analysis suggested that upland tetraploid arose from upland octoploid. All together, this study provides unparalleled insights into the diversity, genomic complexity, population structure, phylogeny, phylogeography, ploidy, and evolutionary dynamics of switchgrass.

## Author Summary

Recent advances in sequencing technologies have enabled large-scale surveys of genetic diversity in model species with a wholly or partly sequenced reference genome. However, thousands of key species, which are essential for food, health, energy, and ecology, do not have reference genomes. To accelerate their breeding cycle via marker assisted selection, high-throughput genotyping is required for these valuable species, in spite of the absence of reference genomes. Based on genotyping by sequencing (GBS) technology, we developed a new single nucleotide polymorphism (SNP) discovery protocol, the Universal Network-Enabled Analysis Kit (UNEAK), which can be widely used in any species, regardless of genome complexity or the availability of a reference genome. Here we test this protocol on switchgrass, currently the prime energy crop species in the United States of America. In addition to the discovery of over a million SNPs and construction of high-density linkage maps, we provide novel insights into the genome complexity, ploidy, phylogeny, and evolution of switchgrass. This is only the beginning: we believe UNEAK offers the key to the exploration and exploitation of the genetic diversity of thousands of non-model species.

## Introduction

In the past decade, switchgrass (*Panicum virgatum* L.) has been targeted as a prime candidate energy crop. As a C_4_ grass, switchgrass has high biomass production with minimal field-based inputs. Its adaptability allows it to be grown productively in large areas of the USA, including marginal lands. In addition, propagation by seed and the perennial growth habit of switchgrass enable relatively effortless establishment, field management and harvest. Although switchgrass shows great promise as a bioenergy feedstock, it would never be considered a model species for genetic or genomic research. Most of the fundamental characteristics of its biology render switchgrass a difficult taxon for the genetic dissection of even the simplest of its useful biofuel-related traits. Switchgrass is a largely self-incompatible and highly heterozygous species [1]. In contrast to species with inbred lines, both forward and reverse genetics are difficult to conduct in switchgrass. In addition, there is evidence of extensive chromosome-number variation, including multiple ploidy levels, as well as aneuploidy [2]. Moreover, switchgrass has a relatively large genome size [2], [3] and lacks a reference genome, both of which hamper the development of an effective marker system. Overall, these challenges are not unique to switchgrass: there are thousands of key species with similar characteristics, and we need tools that can be applied to all of them.

Many of the challenges posed by switchgrass can be overcome through genotyping by sequencing (GBS). This protocol is a multiplexed, high-throughput, and low-cost method to explore the genetic diversity in populations [4]. It employs a reduced representation library (RRL) strategy [5] to target a fraction of the genome for sequencing, thereby decreasing cost and increasing the SNP-calling accuracy. GBS is the simplest of the RRL approaches developed thus far [6]–[9], and has already seen extensive application in a wide diversity of taxa, i.e., in barley and wheat [10], as well as, maize [4], [11], rice, grape and cacao (many publications in progress).

Currently, the RRL strategy has been used for diversity evaluation in various species, resulting in the discovery of hundreds of thousands of SNPs. In most of these cases, the libraries were sequenced on the Illumina platform, and the SNP calling relied on having a reference. The reference could be a high-quality genome sequence [12]–[16], de novo assembly from deep sequencing [17]–[20] or transcriptome sequences [21]. The reference (ideally a reference genome) not only physically orders the SNPs, but also provides the sequence context for paralogs, assigning them to different sites. This reduces the false SNP calls from paralogs, especially in wholly or partly duplicated, or transposon-saturated genomes. However, in the absence of a reference genome, SNP calling may be much less accurate with short-read sequencing technologies, because true SNPs, sequencing errors and SNPs between paralogs can be difficult to distinguish. The Illumina platform and Roche GS-FLX are an effective combination to call SNPs when lacking a reference genome [17]–[20], but additional labor, time and cost are required to build a rough reference with GS-FLX. Therefore, we designed a universal and unconditionally reference-free SNP calling approach to analyze short sequence data from RRLs of any species, especially for the majority which lack a reference genome.

To enable genome-wide association studies (GWAS) and genomic selection (GS) in switchgrass, we developed both linkage and association populations. Phenotypic data from these populations were collected over three field seasons. All 840 individuals in the linkage and association populations were genotyped with GBS. To overcome the inherent difficulties of the lack of a reference genome, multiple ploidy levels and high heterozygosity, a bioinformatics pipeline for SNP discovery based on a species-wide network approach called the Universal Network-Enabled Analysis Kit (UNEAK) was developed. This pipeline was validated in maize and then successfully applied to switchgrass GBS data. High density SNPs were generated to enable future GWAS and GS. Further analysis of the SNP data sets provided unparalleled insights into the diversity, genomic complexity, population structure, phylogeny, phylogeography, and evolutionary dynamics of switchgrass.

## Results

### Development of UNEAK (Universal Network Enabled Analysis Kit), a universal SNP–calling pipeline

**Figure 1:**
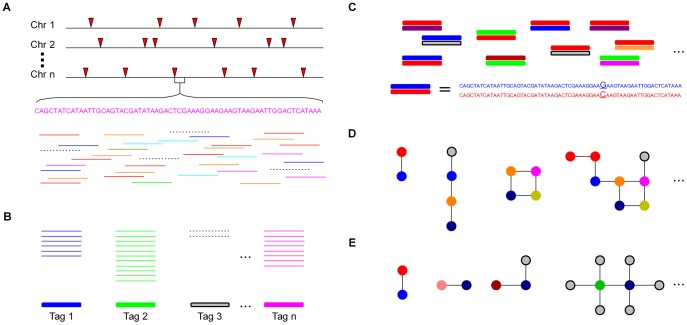
The analytical framework of UNEAK. (A) Multiple DNA samples are digested and sequenced using GBS (red arrows represent cut sites). The inputs of UNEAK are Illumina Qseq or Fastq files. All of the reads are computationally trimmed to 64 bp. The solid colored lines represent error-free (“real”) reads, while the dashed lines are reads containing one or more sequencing errors. (B) Identical reads are classified as a tag. The colored bars are real tags, whereas the shaded bar is a rarer error tag. (C) Pairwise alignment is performed to find tag pairs differing by only a single bp mismatch. (D) Topology of tag networks. The colored circles are real tags. The shaded circles are error tags. Lines (“edges”) are drawn only between tags that differ by a single bp mismatch. (E) Only reciprocal, real tag pairs are retained as SNPs.

**Figure 2:**
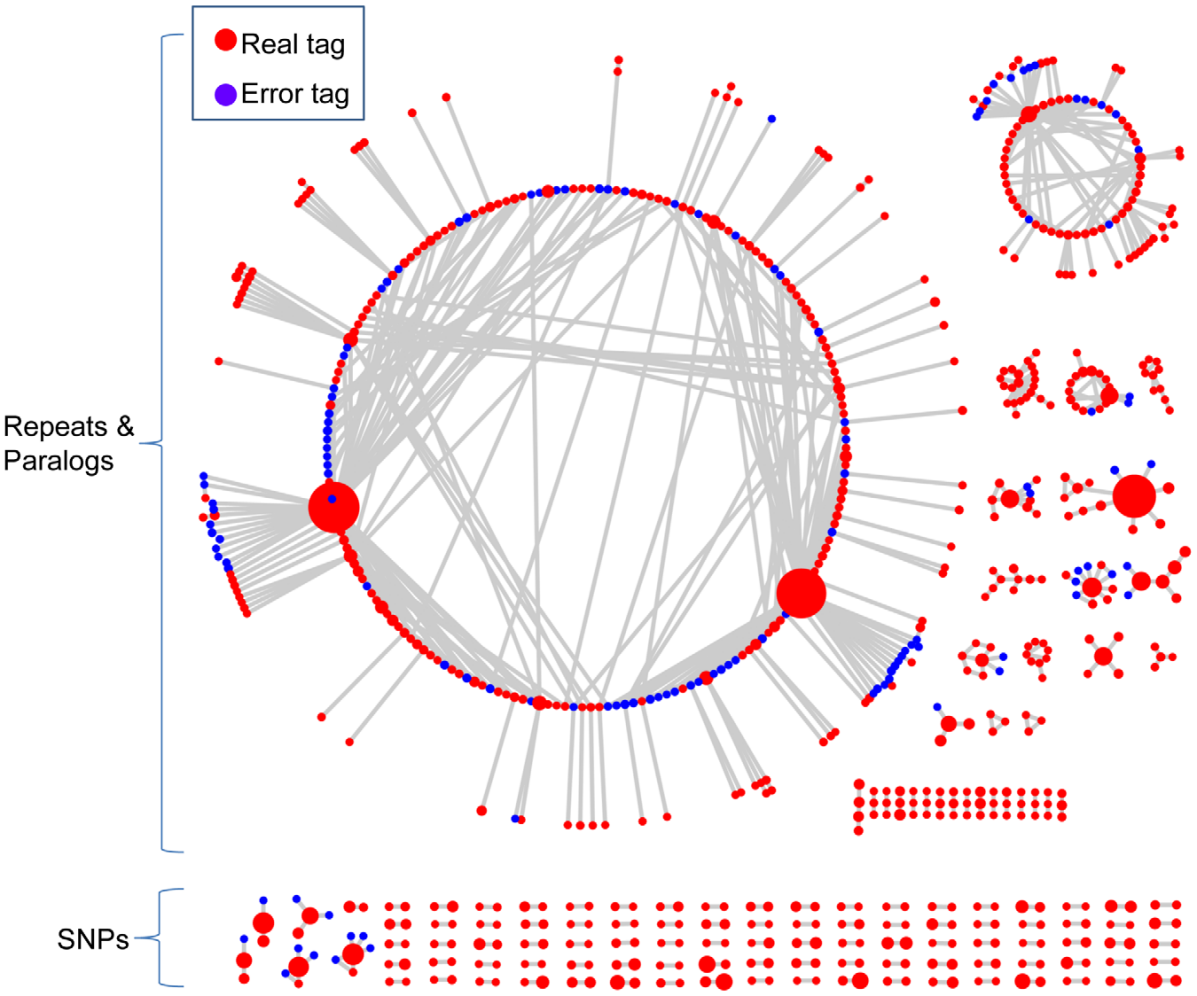
The networks of 802 representative tags from actual switchgrass data. The red circles are putative “real” tags. The blue circles are low frequency, putative error tags (see Methods). The size of each circle denotes the count of a tag. Lines connecting the circles (“edges”) join tags that differ by a single bp mismatch. Of the 802 tags, 192 (24%) formed reciprocal tag pairs and thus, were identified as SNPs by the network filter.

When a reference genome is available, SNP discovery can be easily performed by aligning reads to the physical map. However, when there is no reference genome, as is the case for the majority of species, significant challenges arise. The UNEAK pipeline overcomes many of these challenges. The general design of UNEAK is as follows (Figure 1): Reads are trimmed to 64 bps. The trimmed parts of the reads are ignored because the sequencing errors are enriched at the ends of reads. Identical 64-bp reads are collapsed into tags. Pairwise alignment identifies tag pairs having a single base pair mismatch (Figure 1C). These single base pair mismatches are candidate SNPs. Because of the complexity of the genome, many of the tag pairs form networks (Figure 1D and Figure 2). A network filter is employed to discard complicated networks, which are usually a mixture of repeats, paralogs and error tags (Figure 1E and Figure 2). Ideally, after application of the network filter, the only networks remaining are composed of reciprocal tag pairs, which can then be used for SNP calling.

To account for sequencing errors, we introduced a parameter called the error tolerance rate (ETR) to improve our initial network filter (see Methods). Without this feature (ETR = 0), sequencing errors can have a substantial negative impact upon the number of retained SNPs, especially when the depth of coverage is high. When sequencing errors occur and error detection is not employed, affected tag pairs are no longer reciprocal and therefore are removed from the data set (Figure 1E). By employing an appropriate ETR, the edges between error tags and real tags are cut. In this manner, complicated networks can be separated into different sub-networks, and only those sub-networks composed of reciprocal real tag pairs are kept (Figure S1). Hence, the SNPs with higher coverage, the most valuable part of the data set, are more likely to be retained (Figure 1E and Figure 2).

### Validation of UNEAK using maize GBS data

To validate the UNEAK pipeline, we tested it with GBS data from a single RIL family (B73×B97) from the maize nested association mapping (NAM) population [22]. The large and complex genome of maize [23] makes this a useful test. The 199 inbred lines were processed using the GBS protocol applied in switchgrass. The only difference was that the maize samples were sequenced on the Illumina Genome Analyzer which has about 10% of the throughput of an Illumina Hiseq 2000.

To evaluate the effectiveness of the network filter, we ignored the existence of the maize B73 reference genome and called SNPs at two stages in the pipeline, before and after application of the network filter. The first data set had 336,020 SNPs, which were composed of all tag pairs with a 1 bp mismatch. The second data set was comprised of the 92,951 SNPs that passed the network filter. Only 23.3% of the SNPs in the first data set aligned to a unique site in the maize reference genome. In contrast, after application of the network filter, 78.6% of the SNPs aligned to unique positions. Here, for a uniquely mapped SNP, one tag had a single perfect match to the reference; the other had a single best match at the same site. For the other 21.4%, either or both tags of a SNP aligned equally well to more than one site. Among them, 48.6% SNPs aligned to two sites. Considering that *Ape*KI is a partially methylation sensitive enzyme [4], and that potential tags from long restriction fragments are generally absent from GBS data due to PCR bias, some of the tags that aligned to multiple sites in fact may have come from a single site. To quantify this effect, we performed an *in silico Ape*KI digest of the maize reference genome and identified 8,420,424 potential B73 GBS tag loci. All of the 6,994,161 tags from the B73×B97 family were then aligned to the reference genome; 2,966,692 of these matched perfectly, and thus were B73 tags. These B73 tags accounted for only 35.2% (1,045,475) of the potential B73 tags from the *in silico* digest. Hence, there is a strong possibility that a large proportion of the 21.4% of SNPs from reciprocal tag pairs that align to multiple positions in fact derived from a single genomic position. For example, those SNPs that aligned to two sites (10.4% of total SNPs) had only a 35.2% chance of originating from two genomic positions. Therefore, we estimated that the SNPs essentially aligned to unique positions should be greater than 85%.

The marked difference between the allele frequency distributions before and after application of the network filter demonstrates that this filter substantially improves the quality of the data. In contrast to the pre-network filter distribution, in which only low and high frequency error peaks were discernible, the post-network filter distribution was dominated by a central peak around the expected allele frequency of 0.5. At the same time, the two error peaks located at the two ends of the distribution were significantly reduced (Figure S2).

The 92,951 SNPs were also validated by both linkage disequilibrium (LD) analysis and sequence alignment. First, we calculated LD (*r* ^2^) between these SNPs and the 1106 Illumina Golden Gate SNP markers developed in NAM [22], based on the assumption that valid SNPs should be in LD with adjacent markers. For the 20,402 SNPs with call rates >0.3, the average *r* ^2^ with the four adjacent markers were calculated. The results showed that 92.8% of the GBS SNPs were in LD with a flanking NAM SNP with an *r* ^2^ greater than 0.2 (Figure S3). Second, we aligned the non-B73 tags of these SNPs to the B97 whole genome shotgun sequences from maize HapMap2 data [24], which were sequenced at 4.2× and supposedly covered the majority of the B97 genome. The results showed that 93.2% of the GBS SNPs corresponded to HapMap2 SNPs from B97.

### SNP discovery in switchgrass

To enable GWAS and GS in switchgrass, we created a full-sib linkage population (*n* = 130), a half-sib linkage population (*n* = 168) and an association panel (66 diverse populations, *n* = 540) (Table S1 and Table S2). Using GBS, approximately 350 Gb of sequence were generated from an Illumina HiSeq 2000. The UNEAK pipeline called 400,107, 476,005, and 700,236 SNPs from the full-sib, half-sib, and association populations, respectively. All together, about 1,242,860 putative SNPs were discovered in switchgrass. All of these SNPs had minor allele frequencies (MAFs) greater than 0.05. There were 29,838 (6.9%), 69,605 (12.8%) and 112,099 (13.8%) SNPs with MAFs less than 0.05 in the three populations, respectively. Because we cannot distinguish low frequency SNPs from sequencing errors, SNPs with a MAF less than 0.05 were removed from further analysis. The average coverage of the three data sets was less than 1×, but for some SNPs the coverage was as greater than 6× (Figure S4). The SNP calls can be found at http://www.maizegenetics.net/snp-discovery-in-switchgrass.

### Tetraploid switchgrass behaves like a diploid

The parents of the full-sib population are upland tetraploids. In general, a stretch of DNA sequence should have four orthologous copies in tetraploids. Therefore, when considering the allele frequency distribution of an F1 population, we expected to see seven peaks, representing all possible allele frequency ratios of two parents (e.g., 1∶7, 2∶6, 3∶5, etc.). However, only three peaks were observed (1∶3, 1∶1 and 3∶1) after the network filter was implemented (Figure S2D), the signature of an F1 population of a heterozygous diploid. From this, we infer that tetraploid switchgrass is thoroughly diploidized.

**Figure 3:**
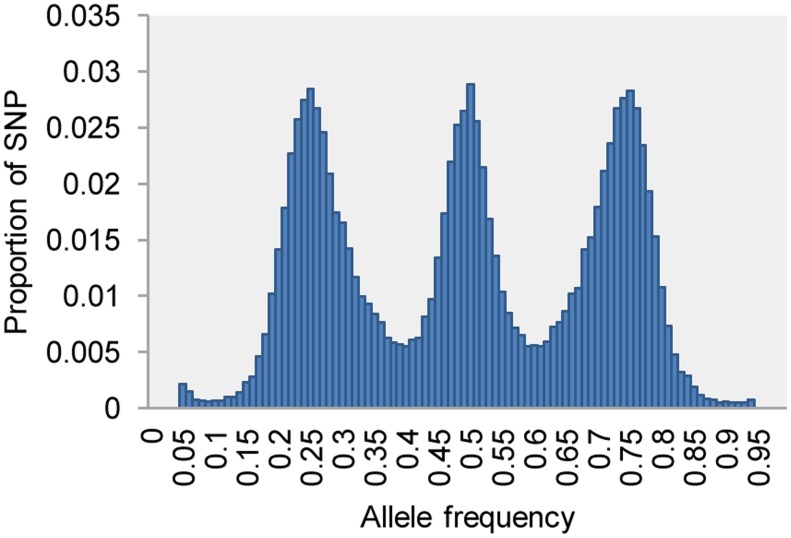
Allele frequency of 50,000 SNPs (call rate >0.8) in the full-sib F1 population (n = 130) of upland tetraploid switchgrass, showing the classic signature of a cross between two heterozygous diploids.

After the network filtering step, a second filter is implemented in UNEAK to remove remaining sequencing errors and paralogs. This filter is a goodness-of-fit χ^2^ test (α = 0.05) based on the null hypothesis that, in diploid species, the counts of the two paired tags of a SNP are equal in all heterozygous individuals. A substantial number of incorrect SNP calls were removed from the data set of the F1 full-sib population (compare Figure S2D to Figure 3). The three peaks of the allele frequency distribution for the remaining SNPs (Figure 3) represent the crosses of AA×Aa (expected allele frequencies of 0.25 and 0.75, with 1∶1 segregation of AA and Aa genotypes), AA×aa (no segregation), and Aa×Aa (expected allele frequencies of 0.5 with genotypic segregation ratios of 1∶2∶1).

The diploid nature of tetraploid switchgrass can also be recognized in individual plants. The tetraploid parents of the full-sib linkage population, U518 and U418, were sequenced at a high coverage of 6×. We ran UNEAK to call SNPs from loci that were heterozygous in both parents. The results showed that the two alleles at heterozygous loci have equal read frequencies within each tetraploid of 0.5 (Figure S5A and Figure S5B), providing more evidence that tetraploid switchgrass is diploidized. To compare the distribution pattern of octoploid and tetraploid, we sequenced one octoploid switchgrass, K101, also at 6×. In contrast to the tetraploids, the read frequency distribution within K101 had three peaks, at 0.25, 0.5 and 0.75 (Figure S5C). This result indicated that octoploid switchgrass behaves more like an autotetraploid.

### Eighteen linkage groups perfectly match the chromosome number of tetraploid switchgrass

GBS is a low coverage genotyping approach, especially when the genome is digested with *Ape*KI, which is a frequently cutting restriction enzyme. Before constructing a high-density linkage map, we first evaluated the quality of the switchgrass genotypic data set. The low depth of sequencing, relative to the number of restriction fragments within GBS size range, has two effects on genotypic data quality. The first is a large amount of missing data. The SNP call rate increases with coverage (Figure S4). Across the 400,107 markers discovered in the switchgrass full-sib linkage population, we achieved a median coverage of 0.54×, which translated into a median SNP call rate of 40%. The second effect of low coverage is that heterozygous SNPs can be miscalled as homozygotes, even at markers with high call rates. To quantify the rate of miscalled heterozygotes, we selected markers with expected allele frequency ratios of 1∶1 (MAF>0.45 in the full sib progeny) that appeared, based upon high coverage GBS data from the parents, to be homozygous in both parents (AA×aa). These markers should be heterozygous in all of the full-sib progeny. As expected, the proportion of miscalled heterozygotes is very high at low coverage markers and declines substantially as coverage increases (Figure S6). In the subset of markers with the highest coverage (>4× coverage, or >90% SNP call rate) we estimate that <30% of heterozygotes were miscalled as homozygous.

**Figure 4:**
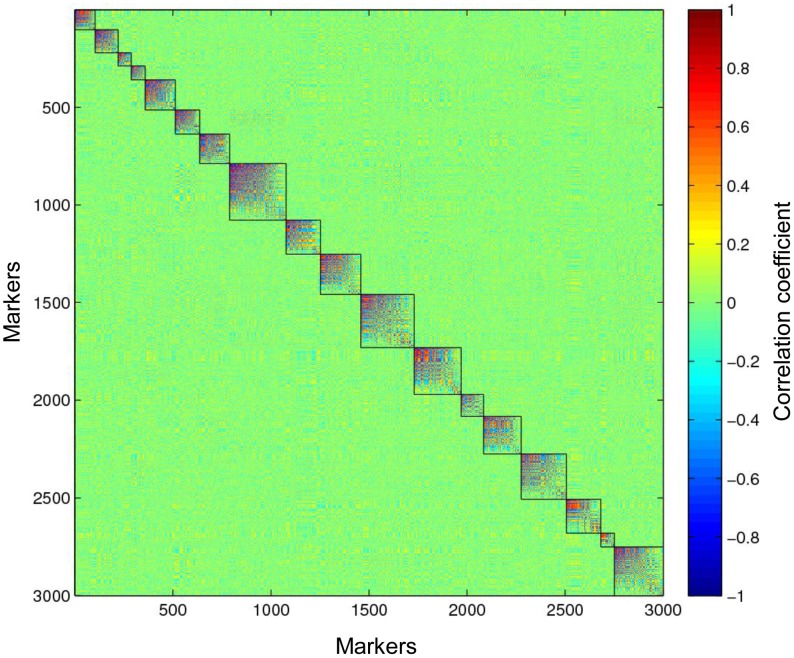
Eighteen paternal linkage groups identified in the full-sib tetraploid linkage population. Three thousand markers are clustered into 18 linkage groups, matching the haploid number of chromosomes in switchgrass. The color scale represents the Spearman's rank correlation between markers.

Due to the large amount of missing data and miscalled heterozygotes, traditional methods to detect linkage based on the LOD score might not be applicable. Therefore, we used the modulated modularity clustering (MMC) method [25] to construct linkage groups. Unlike the agglomerative hierarchical clustering methods used in other genetic map software [26], the MMC is a coherent clustering approach seeking objective groups in the data. Because it does not require input parameters to decide the group number, this approach is completely data driven. Consequently, this clustering method is useful for obtaining linkage groups in a species. To construct linkage groups, we only used the most informative markers (0.2<MAF<0.3) that should be heterozygous in only one of the parents. A subset of these markers, specifically two sets of 3,000 SNPs with a call rate >0.9, or >4× coverage (Figure S4) and with <30% miscalled heterozygotes (Figure S6), were selected for constructing paternal and maternal linkage groups, using the pseudo-testcross [27] mapping strategy (Figure S7). The MMC method was used to group markers based on the Spearman's rank correlation coefficient (r) between marker pairs. This method clustered 3,000 paternal SNPs into 18 linkage groups, which perfectly matches the haploid chromosome number of tetraploid switchgrass (Figure 4). Using the same method, the 3,000 maternal SNPs clustered into 19 linkage groups (Figure S8A). Based on their synteny to foxtail millet (see next section), two of these linkage groups were subsequently merged.

### Synteny-based linkage maps

The next objective of this research was to use pairwise r^2^ to order the markers within each of the linkage groups. This is an example of the travelling salesman problem (TSP), with the additional complication of missing data and that a proportion of the heterozygotes were miscalled as homozygote as a result of the low depth of coverage. We tried several combinatorial optimization methods (e.g. the genetic [28] and ant colony [29] algorithms) to find the optimal order, but none of these resulted in a reasonable marker order. Ultimately, however, we were able to order these SNPs based on the synteny of switchgrass with other grasses, since the grass family has a remarkably conserved genome [30].

Foxtail millet (*Setaria italica*) is the closest relative of switchgrass with an available reference genome (490 Mb) [31]–[33]. It is estimated to have diverged from switchgrass roughly 3–7 million years (Myr) ago and is a diploid species with nine haploid chromosomes, half of the haploid chromosome number of tetraploid switchgrass. We hypothesized that tetraploid switchgrass was formed by a genome duplication after its divergence from the common ancestor (*n* = 9) of the two species. Thus we expected that each of the chromosomes of foxtail millet should align with two linkage groups of switchgrass.

**Figure 5:**
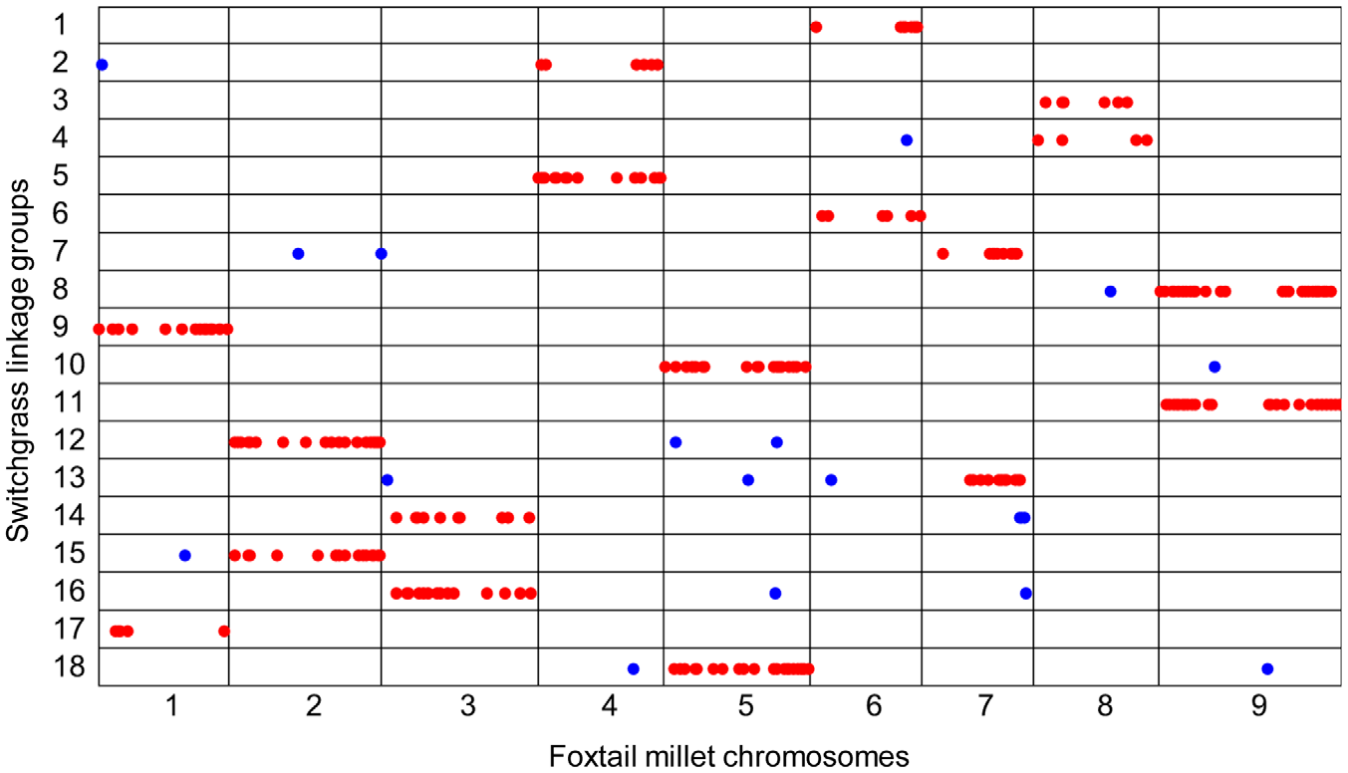
Sequence alignment of SNPs in switchgrass paternal linkage groups to the foxtail millet genome. Nearly 10% (299/3000) of the SNPs previously mapped to switchgrass linkage groups were also mapped to unique sites in the foxtail millet genome. For each linkage group, the majority of SNPs that aligned to one chromosome of the foxtail millet genome are labeled red; the few exceptions are in blue. Each foxtail millet chromosome matches two switchgrass linkage groups, clearly indicating a genome duplication.

By aligning the 3,000 markers in the 18 paternal linkage groups, we found that 299, or nearly 10%, mapped to unique locations in the foxtail millet genome. As expected, the linkage groups of switchgrass matched very well with chromosomes of foxtail millet, indicating that the original linkage group clustering in switchgrass was correct (Figure 5). This result also indicated that strong synteny has been maintained between the two species, in spite of the genome duplication event. Similarly, 339 out of 3,000 (11.3%) markers in the 19 maternal linkage groups also aligned to the foxtail millet genome. In most cases, each foxtail millet chromosome matched two linkage groups, except for chromosome 1. This chromosome had three matches to switchgrass linkage groups. Specifically, linkage groups 1 and 3 were aligned to two separate parts of chromosome 1 (Figure S8B). We hypothesized that the two linkage groups represented one chromosome, but were not successfully clustered together using MMC. Therefore, we merged maternal linkage groups 1 and 3, and thus both the paternal and maternal markers formed 18 linkage groups.

To make high density linkage maps, we used the 6,000 markers from 36 linkage groups (18 paternal linkage groups and 18 maternal linkage groups) as the seed and then attempted to fit as many SNPs as possible into these groups. However, the large proportion of missing data may have a major impact on the clustering. Therefore, markers with call rates of 0.2, 0.5 and 0.9 were used to check the clustering quality (Table S3). Via alignment to the foxtail millet genome, the uniquely aligned markers were identified and clustered into the 36 linkage groups based on the Spearman's rank correlation coefficient. For the data sets with call rates of 0.2, 0.5 and 0.9 respectively, the results showed that 60.2%, 76.8% and 90.8% of the SNPs aligned to physical chromosomes that were syntenic to their linkage groups. Assuming that 90.8% represents the actually degree of synteny conservation between foxtail millet and switchgrass, then it appears that 30% and 14% of the SNPs from the data sets with call rates of 0.2 and 0.5 were assigned to the wrong linkage group, respectively.

To strike a balance between the quality and number of SNPs, the 88,217 SNPs with MAFs between 0.12 and 0.38 and call rate >0.5 were chosen to add to the 36 linkage groups to construct high density linkage maps. Out of these 88,217 SNPs, 9,437 could be aligned to unique positions in the foxtail millet genome; physical and genetic chromosomal assignments agreed for 7,245 of these 9,437. Based on the strong synteny between switchgrass and foxtail millet, we were able to order the 7,245 uniquely aligned SNPs, resulting in paternal and maternal framework maps consisting of 3,244 and 4,001 ordered markers, respectively. To check the quality of the synteny based order in the framework maps, we calculated the pairwise Spearman's rank correlation coefficients for the markers. High coefficient values were distributed along the diagonal in the heat map (Figure S9). This indicates that the synteny between switchgrass and foxtail millet is high enough to provide a reasonable order for switchgrass SNPs. The remainder of the 88,217 SNPs was then placed on the framework maps according to the r^2^ within their assigned chromosomes. A paternal linkage map (18 chromosomes, 41,709 markers) and a maternal map (18 chromosomes, 46,508 markers) were constructed. Both framework maps and the high density linkage maps can be found at http://www.maizegenetics.net/snp-discovery-in-switchgrass.

### Phylogenetic groups reflect ecotype, ploidy level, and geographic distribution

In addition to the genomic analysis of the bi-parental populations, phylogenetic analysis was performed using the SNPs discovered in the diverse association panel. We selected all of the markers with call rates greater than 0.5 in 540 individuals, which included both tetraploid (4×) and octoploid (8×) plants. Because the size of the 8× genome is approximately twice the size of the 4× genome, the SNPs may be biased towards 8× specific SNPs. Furthermore, the octoploid plants may have half the sequencing depth of the tetraploids. Both of these factors have the potential to affect the phylogeny reconstruction. Hence, this data set was evaluated for ploidy specific SNPs as well as for coverage of 4× and 8× switchgrass. Based upon a χ^2^ test, 2.4% and 6.6% of the SNPs had a significantly larger number of genotype calls in 4× and 8× switchgrass, respectively (*p*<0.05), which is similar to the expected type I error rate of 5%. Moreover, the sequencing depth for the 4× and 8× plants was similar, specifically 1.60× and 1.55× coverage for the 4× and 8× switchgrass, respectively. This analysis indicated that the SNPs were suitable for phylogenetic analysis across different ploidy levels.

Using 29,221 markers with call rate greater than 0.5, a neighbor- joining (NJ) tree was constructed based on the pairwise genetic distance among the 540 individuals (Figure S10). To avoid the problem of ploidy specific SNPs mentioned above or the different amount of missing data in individuals, only the sites having genotype calls on both individuals were used while calculating pairwise distance. The phylogeny showed that the upland and lowland ecotypes were clearly separated, with further geographically based subgroups found within each ecotype. Most individuals from the same population were clustered together in the phylogenetic tree.

**Figure 6:**
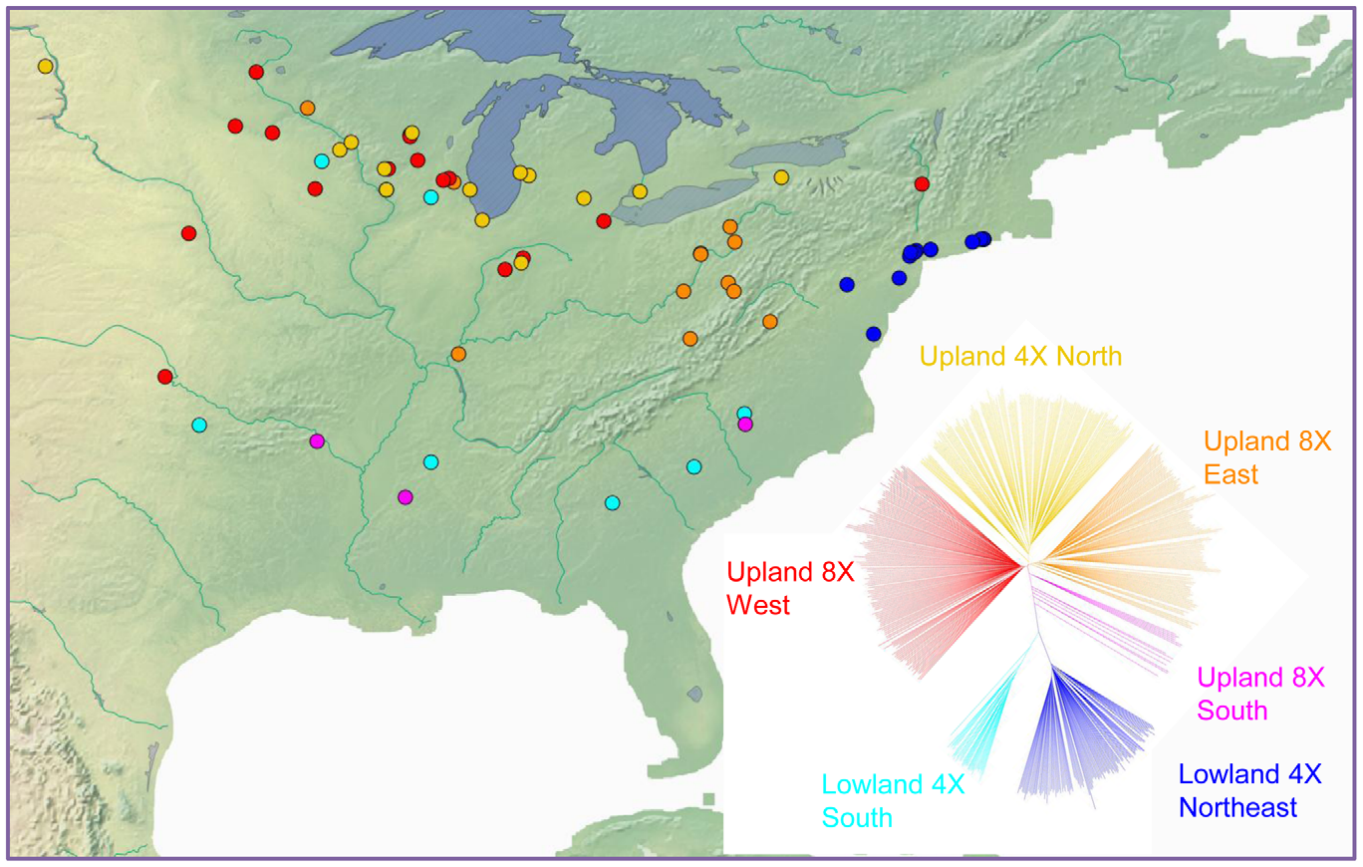
Geographic distribution and phylogenetic groups of switchgrass in the association panel. Each population is indicated by a dot on the map in its approximate source location and a branch in the phylogenetic tree of the same color. Clades are labeled with ecotype, ploidy and geographical descriptors.

Ploidy variation in switchgrass is ecotype-specific: plants of the lowland type are tetraploid, whereas those of the upland ecotype are primarily tetraploid (4×) or octoploid (8×). We estimated ploidy level in at least one clone per population in the diversity set using flow cytometry (Table S1). This ploidy information was mapped onto the marker-based phylogeny of switchgrass, indicating that ploidy level also resolves into distinct groups (Figure S11). Isolation by distance is also clearly indicated by geographic analysis. A Mantel test showed that genetic and geographic distance were significantly correlated (r = 0.51, *P*-value<0.001). A direct comparison of the groups indicated by the phylogeny with their geographic origins (Figure 6) further illustrated the strong influence of geography on the distribution of genetic diversity in this widespread species.

### Evolutionary dynamics

Clearly, the phylogeny of switchgrass concurs well with ecotypes, ploidy level and geographic distribution. However, what does the phylogeny tell us about the evolutionary origin of the upland octoploid? Is it an allopolyploid, formed by a wide hybridization between an upland ecotype 4× and a lowland ecotype 4×? Or is it the product of a combination of two upland 4×, resembling more of an autopolyploid origin? The first scenario is not likely, because the upland 8× is not intermediate between the ecotypes, but more closely related to the upland 4× (Figure 6). To address the second scenario, we first identified an appropriate outgroup.

Foxtail millet, which proved to be highly informative for linkage mapping in switchgrass, was an ideal outgroup for this study. As demonstrated, it is possible to uniquely align approximately 10% of switchgrass SNPs to foxtail millet. Of the 29,221 markers used for the phylogeny analysis, 3,144 aligned to the foxtail millet genome. Comparing these SNPs to the foxtail millet genome, we identified the ancestral alleles of switchgrass and assigned 3.1 kb of foxtail millet sequence as the outgroup. Next, a NJ tree was constructed with 500 bootstrap replicates (Figure S12). Upland and lowland ecotypes were well separated. However, even 3,144 markers were unable to resolve the sub-groups within the upland ecotype with high bootstrap values. Nevertheless, all of the lowland individuals formed one clade, with a bootstrap value of 100%. For the next stage of the analysis, the lowland ecotype was designated as the outgroup for upland switchgrass.

**Figure 7:**
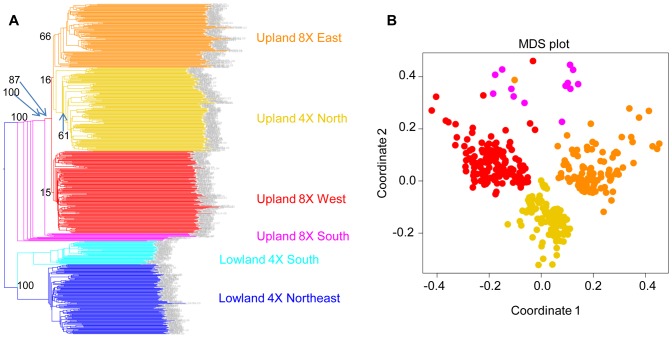
Upland 4× arose from 8×. (A) A NJ tree of 29,221 markers. The lowland clade is the outgroup. (B) Multiple dimensional scaling (MDS) plot of the upland ecotypes.

Taking the lowland ecotype as the outgroup, we bootstrapped the tree based on 29,221 markers (Figure 7A). The results showed that within the upland ecotype, 8× East and 4× North constitute distinct groups. However, the 8× West clade has a low bootstrap value of 15%. We inferred that this is because Upland 8× West group contains admixed individuals that overlap genetically with Upland 8× East and Upland 4× North. Because the Upland 4× North is an inner branch of Upland 8× West, it is unlikely that the upland 4× gave rise to upland 8×.

In fact, our analysis suggests the opposite: upland 4× arose from upland 8×. The upland south clade is the outgroup of three other clades of upland switchgrass with a bootstrap value of 100% (Figure 7A). Further evidence supporting this came from the multiple dimensional scaling (MDS) plot (Figure 7B). The MDS was based on the kinship matrix of individuals of the upland ecotype. The Upland 4× North clade has clearly reduced diversity compared to the upland 8× groups.

To formally test the two competing scenarios, namely of (1) upland 4× arising from upland 8×, versus (2) upland 4× arising from lowland 4×, we constructed an alternate, constrained topology consistent with scenario two (Figure S13). Using the program MEGA, we calculated the likelihood of the two topologies using a random subset of 5,000 out of the original 29,221 markers. The original topology, corresponding to scenario one, was 10^590^ times more likely than the alternate, constrained topology, strongly supporting the scenario where upland 4× arose from upland 8×.

## Discussion

### The development of UNEAK

Genomic selection and GWAS have the potential to substantially improve the efficiency of breeding programs [34]. The decreasing cost and increasing throughput of next generation sequencing have enabled large scale SNP discovery efforts in many species, particularly those that are important in the current agricultural economy, are well-characterized genetically, and already have reference genomes. However, the growing demands for energy and environmental conservation require the breeding of an increasingly diverse set of species. Many of these species, including switchgrass, currently lack reference genomes. While significant gains have been made since the inception of switchgrass breeding in the 1950s [35], genome-based selection methods offer significant opportunities to increase the rate of gain [36].

SNP discovery from next-generation sequencing data is particularly challenging in the absence of a reference genome. Our SNP calling pipeline, UNEAK, was developed specifically in response to this challenge. Unlike most non-reference SNP calling protocols, UNEAK does not require a partially sequenced genome, contigs from additional sequencing platforms [17]–[20], or a transcriptome to serve as a pseudo reference genome [21]. By constructing networks of tags, UNEAK mimics the processes of replication and mutation of paralogous sequences. Filtering out the more complex networks resolves the paralog and repeat issues which hinder SNP discovery efforts in species with large genomes, multiple ploidy levels, or without reference genomes. Starting with high throughput GBS reads from the Illumina platform, UNEAK provides a time- and cost-efficient way to generate hundreds of thousands of markers for population evaluation, linkage map construction, quantitative trait loci (QTL) mapping, GWAS and GS, in species with limited genetic resources. These high density markers will greatly facilitate genomic selection in biofuel species or in species with agricultural, ecological, or medical importance.

Continuing efforts have been made to call SNPs in species lacking a reference genome. For example, the restriction-site associated DNA (RAD) [7] method yields high coverage SNPs using the 8 bp cutter, *Sbf*I, that can be successfully used for phylogeographic study [37] and genetic map construction [38], [39]. However, the RAD analysis pipeline, Stacks [40], requires high coverage sites and assumes that the species under investigation is diploid. In contrast, UNEAK can perform well for both high and low coverage genotyping methods. Moreover, UNEAK can be used in polyploid species, which are becoming more economically important. In addition, the high density panel of SNPs discovered by UNEAK provides an opportunity to conduct GWAS and GS to accelerate the breeding process.

Even though UNEAK was designed for SNP discovery in species without reference genomes, it can also be used for species with a reference genome. In fact, most reference genomes generally do not cover the whole genome of a species, for two reasons. The first is technical: a reference genome derived from a single individual is usually incomplete because of technical difficulties. In other words, some genomic regions are “technically missing”. The second is biological: one individual's genome does not completely represent the whole genome of that species, because of presence and absence (PAV) variation. Regions containing PAVs can be “biologically missing.” In either case, the genetic variation in the missing genome is basically inaccessible when using SNP discovery methods that rely on the reference genome. UNEAK makes it possible to gain access to those missing regions.

### Validation of UNEAK pipeline

In this study, maize was used to validate the UNEAK pipeline. Maize is a large and complex genome, which experienced multiple genome duplication events [41], [42], has a large amount of repetitive sequence, numerous PAVs [23], [43], [44], with only about 50% overlap in sequence content between any two unrelated inbred lines [45], [46]. Results from maize convincingly validated the UNEAK pipeline. Of the 92,951 SNPs discovered by UNEAK, 78.6% aligned to unique positions in the maize reference genome. Because not all potential GBS tag loci in the maize reference genome are accessed by GBS, the actual proportion of unique SNPs should be above 85%. Validation by either LD or alignment suggested that >92% of the SNPs with MAFs greater than 0.05 were legitimate. The other 8% were probably due to sequencing errors or paralogs. However, these false positives can be significantly reduced through use of a minor allele frequency filter in biparental populations. For example, for SNPs with a MAF>0.3, the validation rate reaches 96.2%.

The UNEAK pipeline was designed to perform SNP discovery in a broad range of species, therefore only the network filter, which is the key to UNEAK, was implemented and evaluated in the maize test. Essentially, based on the tag count file output from UNEAK, end users can design filters specific to the biology of their study population. For example, repulsion of alleles in inbred lines, or equal tag count of alleles at heterozygous sites in diploid species can be tested to filter for higher quality SNPs.

Although UNEAK generates reliable SNPs, it cannot guarantee high quality genotype calls when sequencing coverage is low. Coverage has a major impact on genotype quality (Figure S4 and Figure S6). Call rate increases with coverage, but with diminishing returns (Figure S4). In inbred lines or haploid germplasm, especially in species with a reference genome, too much coverage is a waste of sequencing resources. On the other hand, in highly heterozygous germplasm, high rates of coverage may be required to distinguish heterozygous sites from homozygous, depending on the desired level of error tolerance. In this case, to obtain high quality genotypes from GBS, it may be helpful to use enzymes with longer recognition sequences than *Ape*KI.

### Linkage map construction using GBS

A high quality linkage map is essential for QTL mapping and the assembly of whole genome sequence. The average coverage of the SNPs in the switchgrass full-sib population is only 0.95×. Even so, we were able to identify linkage groups. Using the 3,000 SNPs with the highest call rate (>0.9), 18 linkage groups were successfully identified and confirmed by alignment to the foxtail millet genome, for both paternal and maternal markers. However, without relying on synteny with foxtail millet, we were unable to order the markers within the linkage groups, even after trying many different algorithms. Our inability to order the markers stemmed from the low sequencing coverage; this led to high amounts of missing data, and heterozygotes often being miscalled as homozygotes, even for SNPs with high call rates (Figure S4 and Figure S6). For the same reason, the high density linkage maps (41,709 SNPs in the paternal map and 46,508 SNPs in the maternal map) we developed based on the lower call rate (>0.5) had approximately 14% markers grouped to wrong chromosomes. This percentage was largely reduced for the SNPs uniquely aligned to the foxtail millet genome and clustered into the one of the syntenic linkage groups of switchgrass. These SNPs comprise the framework maps, which were ordered based on the strong synteny between the two species [33]. Both high density maps (41,709 and 46,508 SNPs) and high quality framework maps (3,224 and 4,001 SNPs) are available at our website. These linkage maps should be useful for the current switchgrass genome assembly effort by the Joint Genome Institute. For example, they can be used to differentiate contigs derived from the two subgenomes as well as to order contigs on a chromosome.

The GBS protocol generally provides low coverage if the enzyme *Ape*KI is used, but higher coverage can be obtained by choosing enzymes with longer recognition sites [4]. There is a tradeoff between coverage and number of SNPs. For linkage map construction, where the number of recombination events is limited, thousands of SNPs usually provide sufficient resolution. However, in breeding applications, a higher density of SNPs should provide a better chance to find SNPs that are tightly associated with QTLs. Therefore, further development of UNEAK will focus on linkage map construction using a six base cutter such as *Pst*I in GBS, which is expected to result in at least 8 times higher coverage than *Ape*KI and thus should provide a better balance between coverage and total number of SNPs.

### The diploidization of switchgrass

Ploidy level variation significantly complicates genetic research in switchgrass. The F1 full-sib population of switchgrass in this study was made by crossing two upland tetraploids. Although seven peaks were expected in the allele frequency distribution in the F1s, the three peaks clearly indicated that the tetraploid switchgrass behaves like a diploid (Figure 3). We also observed nearly equal intra-individual allele read frequencies of ∼0.5 at heterozygous loci within individual tetraploid plants. In addition, the MMC method successfully clustered paternal and maternal markers into 18 and 19 linkage groups respectively, with the extra maternal linkage group later merged with another via synteny. The correlations within the linkage groups are visibly higher than between groups (Figure 4). Construction of an SSR-based linkage map for switchgrass also indicated that chromosomes pair preferentially in meiosis [47]. These four lines of evidence indicate that tetraploid switchgrass shows disomic inheritance and has undergone diploidization over the past one million years [48]. The diploid nature of tetraploid switchgrass will greatly simplify genetic research and whole genome sequencing efforts.

### Population structure, phylogeography, and evolution of switchgrass

Based on 29,221 markers, the phylogenetic analysis in this study provides high resolution to cluster the 540 individuals from 66 populations. As reported by previous studies using either sequence from chloroplast genomes [49]–[51] or SSR markers in nuclear genomes [51], [52], early divergence of the upland and lowland ecotypes was also observed in this study. In most cases, individuals from the same population were grouped together. Additionally, we found distinct subgroups within each ecotype based on their geographic distribution. This result clearly indicates isolation by distance, which could not be detected by random amplified polymorphic DNA (RAPD) markers [53]. The fact that subgroups of different ploidy form distinct clades indicates that the two ploidy levels are reproductively isolated [1].

To investigate if non-random patterns of shared missing genotypes between individuals affected the tree topology, we evaluated the ploidy specific SNPs and coverage of the 4× and 8× switchgrass. Only 6% of the SNPs (slightly higher than the type I error rate) were octoploid specific, and sequence coverage was quite similar in the two ploidy groups. These observations indicate that octoploid switchgrass behaves like an autotetraploid (Figure S5). Hence, it does not contain many private genomic regions relative to tetraploid switchgrass. Moreover, only the sites with genotype calls in both individuals were used to calculate the genetic distance, which should minimize the impact of differential amounts of missing data. In addition, we reconstructed multiple trees at different call rates ranging from 0.15 to 0.9, and the overall topology with respect to the main groups, for example Upland 8× West, Upland 4× North, Upland 8× East, Upland 8× South, Lowland South and Lowland Northeast, was stable regardless of call rate (data not shown). When call rate was greater than 0.9, there were only about 800 SNPs in the data set, many of which were repetitive paralogs. When the call rate is less than 0.15, there were not enough shared sites between individuals to calculate genetic distances. Thus, in spite of the low coverage of GBS, we concluded that the phylogenetic relationship constructed in this study is reliable. These results also suggest that missing genotypes do not alter the performance of phylogenetic analysis, provided that large numbers of SNPs are used.

Our results suggest that the upland 4× arose from upland 8×. We used a stepwise method to designate the lowland ecotype as the outgroup of the upland ecotype. Phylogenetic analysis indicated the upland 8× is closer to this external, lowland branch. Additionally, upland 4× showed less diversity than upland 8×. Both lines of evidence support the hypothesis that upland 8× gave rise to upland 4×. This conclusion is contradictory to the accepted evolutionary trajectory of higher ploidy level being derived from either auto- or allo-polyploid events involving lower ploidy taxa. A reversion to the lower ploidy could occur via apomixis, whereby an unfertilized haploid (4×) gamete becomes a viable embryo. This is a well-documented phenomenon in perennial grasses [54]. Confirmed haploidy of switchgrass has been observed in two laboratories, in both cases at extremely low frequencies, on the order of 10^−4^ to 10^−2^, from the 2n = 4x = 36 to the 2n = 2x = 18 ploidy level [49], [55]. The relatively high frequency of tetraploid accessions in the northern USA (Figure 6) cannot be explained simply by this phenomenon, suggesting that selection may play a role in favoring upland tetraploid genotypes in certain northern environments.

According to the phylogeny (Figure 7) and the geographic distribution of upland switchgrass (Figure 6), we confirmed a south to north migration path of upland switchgrass, which agrees with a previous study [51]. Our data also indicated a loss of diversity during the migration, manifested largely by the shift in ploidy from 8× to 4× in the north. For the lowland switchgrass, our phylogenetic analysis cannot tell the migration direction. However, it is very likely that the common ancestor of upland and lowland ecotypes came from the southern area, then migrated to the north, not vice versa. Therefore, we inferred a general south to north migration path of switchgrass (Figure S14). The natural barrier of the Appalachian Mountains split the northern spread of switchgrass into two subgroups. To the west of the mountains, the most recent common ancestor (MRCA) of the Upland ecotype was 8×. During migration to the north, a ploidy level shift occurred and the Upland 4× emerged. To the east of the Appalachian Mountains, the lowland ecotype was favored, and continued spreading northward along the coastal plains. Subsequently, the Lowland 4× Northeast subgroup diverged both geographically and genetically from the Lowland 4× South.

The switchgrass germplasm used for this phylogenetic study derived mainly from northern-adapted populations, with very restricted sampling of populations from southern and central regions. Based on the geographic distribution and deep divergence of the upland and lowland ecotypes [51], [52], we expect the switchgrass from southern regions, particularly in Mexico, Texas and Florida, to be clustered with the lowland ecotype. Switchgrass from the central regions might provide useful information about the divergence of switchgrass into two ecotypes, the origin of upland ecotypes, and how the ploidy level shifted during the migration. More extensive sampling of switchgrass from all regions of North America will undoubtedly improve our understanding of switchgrass evolution.

## Materials and Methods

### Switchgrass germplasm

The association panel consisted of 66 diverse switchgrass populations grown from seed in the greenhouse of the USDA-ARS Dairy Forage Research Center in Madison WI in 2007. This panel was mainly composed of northern adapted upland populations (Table S1). In addition, two tetraploid F1 linkage populations were propagated at the same time. One was derived from the bi-parental cross of two upland accessions from the germplasm collection of MDC, named U518 and U418. The other was a half-sib population whose maternal parent is U601. The numbers of individuals in these two populations were 130 and 168, respectively. For the association panel, ten clones from each population were initially planted in replicated field plots in Ithaca, NY and Arlington, WI in 2008. All genotyping was conducted on plants from the Ithaca site.

### Reduced representation libraries construction and sequencing

The reduced representation libraries were constructed and sequenced according to the published GBS protocol [4] with one modification. Specifically, a titration experiment showed that ∼0.1 pmol of each adapter was appropriate for switchgrass (rather than ∼0.06 pmol), and that amount was used with 100 ng of genomic DNA. DNA samples were digested with the restriction enzyme *Ape*KI, which has a 4.5 bp cut site (CGWGC, where W = A or T). The resulting libraries were sequenced on the Illumina HiSeq 2000. Ninety five samples (plus a blank negative control) were sequenced per lane.

### SNP discovery and genotyping

The non-reference pipeline UNEAK (http://www.maizegenetics.net/gbs-bioinformatics) was developed for SNP discovery and genotyping in species like switchgrass (Figure 1). In UNEAK, Illumina reads are trimmed to 64 bp and stored in bit format, which greatly reduces the amount of storage space and enables relatively fast computation. About 40GB of data from one lane of an Illumina HiSeq 2000 can be processed to SNP genotypes in 20 minutes on a personal computer with 2.67 GHz CPU and 8 GB memory. More technical details of UNEAK are described in Text S1.

The network filter is the key step for identifying and removing paralogs. The simplest networks, reciprocal tag pairs, are more likely to be real SNPs than tag pairs that are part of complicated networks. Rare tags, with read counts below a specified error tolerance rate (ETR), are assumed to result from sequencing error. For two tags (t1 and t2) with a 1 bp mismatch and read counts c1 and c2, if c1/(c1+c2)<ETR, then t1 is assumed to be a sequencing error of t2. The edges connecting real tags and error tags are then sheared, dividing the network complexes into parts. Remaining reciprocal, real tag pairs are then identified as SNPs (Figure S1).

According to the frequency distribution of tag pairs in structured populations (Figure 2A and Figure S2C), we found the frequencies of most sequencing errors were less than 3%. Therefore, the ETR was specified as 0.03 in this study.

To calculate the coverage in the SNP data sets, we calculated the sum of the read counts of all of the sites across all the individuals. This sum is then divided by the number of sites and number of individuals. So the measurement of coverage in this study is reads/site/individual.

### Linkage map construction

The maternal and paternal parents of the full-sib linkage population, U518 and U418, were sequenced via GBS at about 6×, i.e., at six times higher coverage than the F1 individuals in the full-sib population. SNP markers that were homozygous in one parent and heterozygous in the other and which had a minor allele frequency (MAF) in the progeny between 0.2 and 0.3 were chosen for linkage analysis via the pseudo-testcross mapping approach [27] (Figure S7). We selected two sets of 3,000 markers for paternal and maternal linkage groups, respectively.

Initial linkage groups were constructed based upon the 6,000 markers with the highest call rate (>0.9). The MMC method [25] was used to cluster the markers into linkage groups. In the MMC input file, homozygous genotypes were assigned a value of 0 or 2, and heterozygous genotypes and missing data were assigned a value of 1. Spearman's rank correlation coefficient was used by MMC.

Markers representing the cross of AA×Aa segregate from either a paternal or maternal linkage group. Using the 36 linkage groups produced by MMC from these initial 6,000 markers as seeds, 88,217 markers (0.12<MAF<0.38, call rate >0.5) were then assigned to linkage groups based upon their Spearman's rank correlation coefficient to each seed linkage group.

To order the markers within each linkage group, we relied upon extensive synteny between the switchgrass and foxtail millet genomes. Markers were mapped to the foxtail millet genome (http://www.phytozome.net/foxtailmillet.php) via Basic Local Alignment Search Tool (BLAST) [56] with a *P*-value cutoff of 1e-5. The 7,245 markers that mapped to a single site of the foxtail millet genome, and clustered with one of the syntenic linkage groups of switchgrass, were used to construct the synteny based framework linkage maps. To construct the high density linkage maps, the rest of the 80,972 markers were mapped to the framework marker with highest value of Spearman's rank correlation coefficient within their assigned linkage groups.

### Phylogenetic analysis and evolution

A pairwise genetic distance matrix between individuals was calculated and an un-rooted NJ tree constructed using TASSEL [57]. All of the 29,221 markers with a call rate greater than 0.5 in the diverse populations were used in this analysis. To assess the robustness of the topology of the tree, 500 bootstrap replicates were performed using MEGA [58].

To address the evolutionary trajectory of upland switchgrass, a two-step phylogenetic analysis was performed. In the first step, foxtail millet was used as an outgroup. A NJ tree was reconstructed based on the 3,144 SNPs that could be aligned to unique positions in the foxtail millet genome. This first step identified the lowland ecotype as ancestral to the remaining switchgrass ecotypes studied herein. The second step omitted foxtail millet and used the lowland ecotype as the outgroup. This second NJ tree was reconstructed based on 29,221 markers (alignment to foxtail millet not required). An MDS plot was also generated based upon the kinship matrix of individuals calculated from the 29,221 markers in TASSEL [57].

## Supporting Information

Figure S1 Details of the network filter. The dots represent tags. The size of dots increases with tag count. Blue dots are putative sequencing errors (rare tags). Red dots are real, more common tags. Arrows from the blue dots to the red dots indicate where the errors come from. (A) A network of tags. (B) The sequencing errors are identified if their counts are much fewer than the counts of adjacent tags. (C) The edges connecting the real tags and errors are sheared. (D) The network is divided into sub-networks. The reciprocal tag pair is kept as a potential SNP. The network with multiple tags is discarded. (E) Possible tag topologies of potential SNPs after passing through the network filter.
(TIF)

Figure S2 Effect of the network filter on actual allele frequency distributions in biparental populations. SNPs were called in a single family (B73×B97) from the maize NAM population [22] (A and B) and in a switchgrass full-sib F1 linkage population (C and D). We called SNPs based on finding tag pairs mismatching at a single base (A and C) and then filtered these SNPs with the network filter (B and D). The peaks at the two ends of the distributions correspond to artifactual SNPs with low minor allele frequencies resulting from sequencing errors.
(TIF)

Figure S3 LD distribution of SNPs generated from UNEAK versus 1106 external SNP markers in the maize NAM family B73×B97 [22]. For each UNEAK SNP, the average LD (*r* ^2^) with 4 adjacent, external SNPs was calculated.
(TIF)

Figure S4 The relationship between coverage and SNP call rate in the switchgrass data sets. The call rate represents the proportion of individuals that was covered by at least one read. A total of 3,000 SNPs are plotted in each subfigure. (A) is from the full-sib population (130 individuals). (B) is from the half-sib population (168 individuals). (C) is from the association populations (540 individuals).
(TIF)

Figure S5 Relative depth of coverage (read frequency) of the two SNP alleles at heterozygous loci in the tetraploid U518 (A), the tetraploid U418 (B), and the octoploid K101 (C).
(TIF)

Figure S6 The relationship between sequencing coverage and the proportion of miscalled heterozygous genotypes. A total of 3,000 SNPs are plotted. Due to the limited sequencing depth, heterozygotes are often miscalled as homozygotes.
(TIF)

Figure S7 Marker selection and linkage detection via a pseudo-testcross strategy. The red and blue blocks represent bi-allelic SNPs in each site. (A) Only SNPs with allele frequencies falling within 0.2<MAF<0.3 in the F1 were selected. These SNPs must be homozygous in one parent and heterozygous in the other parent. (B) A subset of SNPs which are all homozygous in one parent and all heterozygous in the other parent, but with unknown linkage phase, were selected. (C) Close linkage of SNPs was detected based upon Spearman's rank correlation. When two SNPs are in coupling phase, the correlation is positive; when they are in repulsion, the correlation is negative. High r^2^ indicates tight linkage.
(TIF)

Figure S8 Maternal linkage groups in a biparental switchgrass family and their alignment to foxtail millet genomes. (A) 3,000 markers were clustered into 19 groups. (B) The 19 groups were aligned to the foxtail millet genome. The majority of the SNPs on each linkage group aligned to the same chromosome of the foxtail millet genome (red dots); there were some exceptions, however (blue dots). In most cases, each foxtail millet chromosome matches two linkage groups of switchgrass. The sole exception, chromosome 1, has three matching linkage groups (1, 3 and 10). Based on later analyses, we merged linkage groups 1 and 3.
(TIF)

Figure S9 Pairwise Spearman's rank correlation coefficient (r) of ordered markers on paternal linkage group 12. A total of 228 markers were ordered based on their alignment to the foxtail millet genome. Pairwise r was calculated for these markers. The high r values were distributed along the diagonal.
(TIF)

Figure S10 Neighbor-Joining tree of 540 individuals from 66 diverse populations of switchgrass. In the tree on the left, the red branches indicate the upland ecotype of switchgrass. The blue branches are the lowland ecotype. Details of a portion of the tree are shown on the right. Individuals within a box are from the same population.
(TIF)

Figure S11 Switchgrass ecotypes with different ploidy levels resolve into distinct phylogenetic clades.
(TIF)

Figure S12 Switchgrass Neighbor-Joining phylogeny constructed with 3,144 markers. Foxtail millet was used as an outgroup.
(TIF)

Figure S13 Two competing evolutionary models of upland 4× switchgrass. (A) Upland 4× arose from upland 8× switchgrass. (B) Upland 4× arose from lowland 4× switchgrass.
(TIF)

Figure S14 Migration patterns of switchgrass.
(TIF)

Table S1 Background information on the germplasm used in study.
(PDF)

Table S2 Individual clones sequenced in the association panel.
(PDF)

Table S3 Quality of linkage group clustering at different call rates.
(PDF)

Text S1 Technical details of UNEAK pipeline.
(DOCX)
