# Supplementary figures and images for: Switchgrass Genomic Diversity, Ploidy, and Evolution: Novel Insights from a Network-Based SNP Discovery Protocol
Source: PLoS Genet. 2013 Jan 17;9(1):e1003215. doi: 10.1371/journal.pgen.1003215 (PMC3547862; doi:10.1371/journal.pgen.1003215)

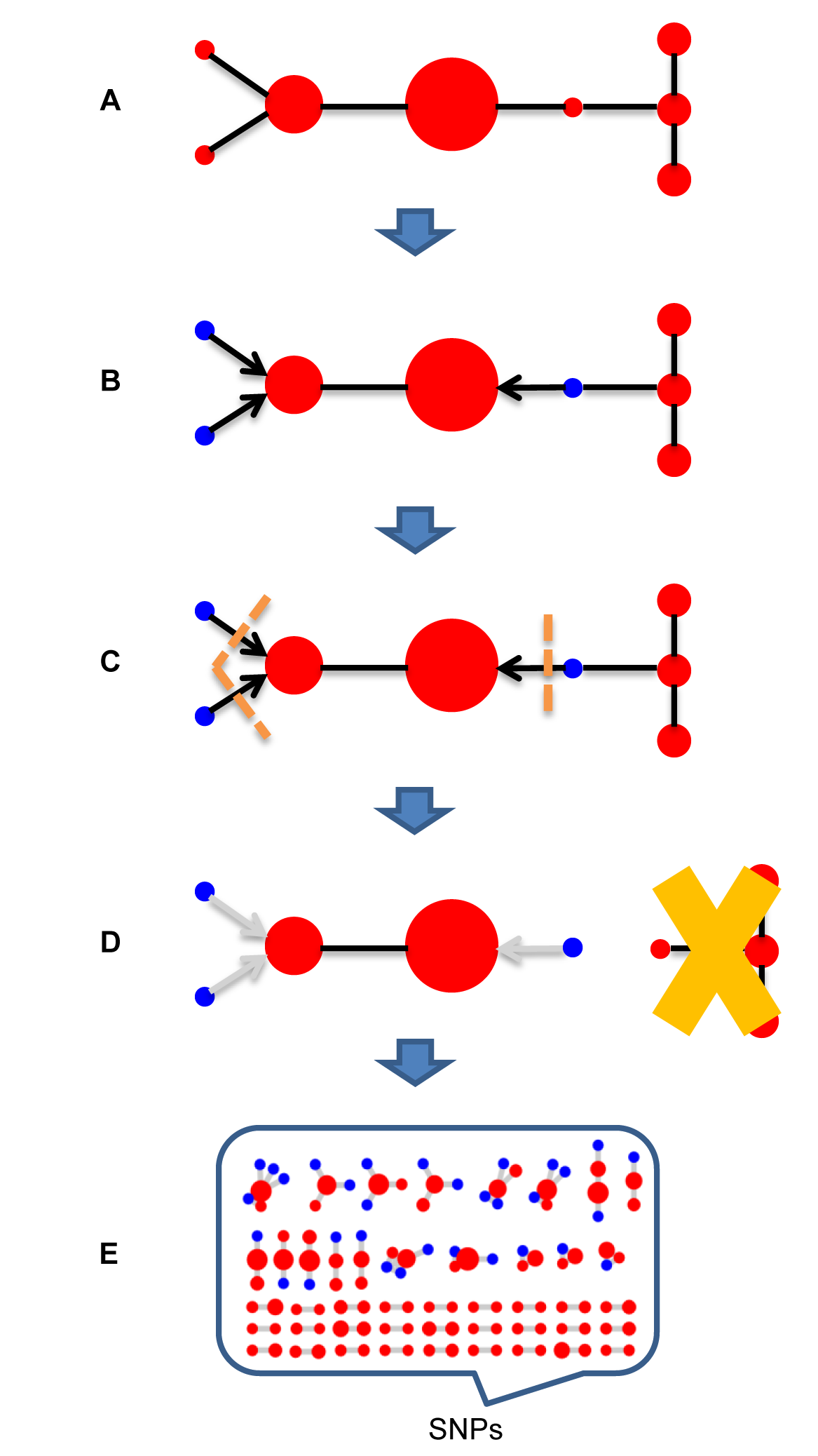

Supplement: Figure S1 — Details of the network filter. The dots represent tags. The size of dots increases with tag count. Blue dots are putative sequencing errors (rare tags). Red dots are real, more common tags. Arrows from the blue dots to the red dots indicate where the errors come from. (A) A network of tags. (B) The sequencing errors are identified if their counts are much fewer than the counts of adjacent tags. (C) The edges connecting the real tags and errors are sheared. (D) The network is divided into sub-networks. The reciprocal tag pair is kept as a potential SNP. The network with multiple tags is discarded. (E) Possible tag topologies of potential SNPs after passing through the network filter. (TIF) [file pgen.1003215.s001.tif]

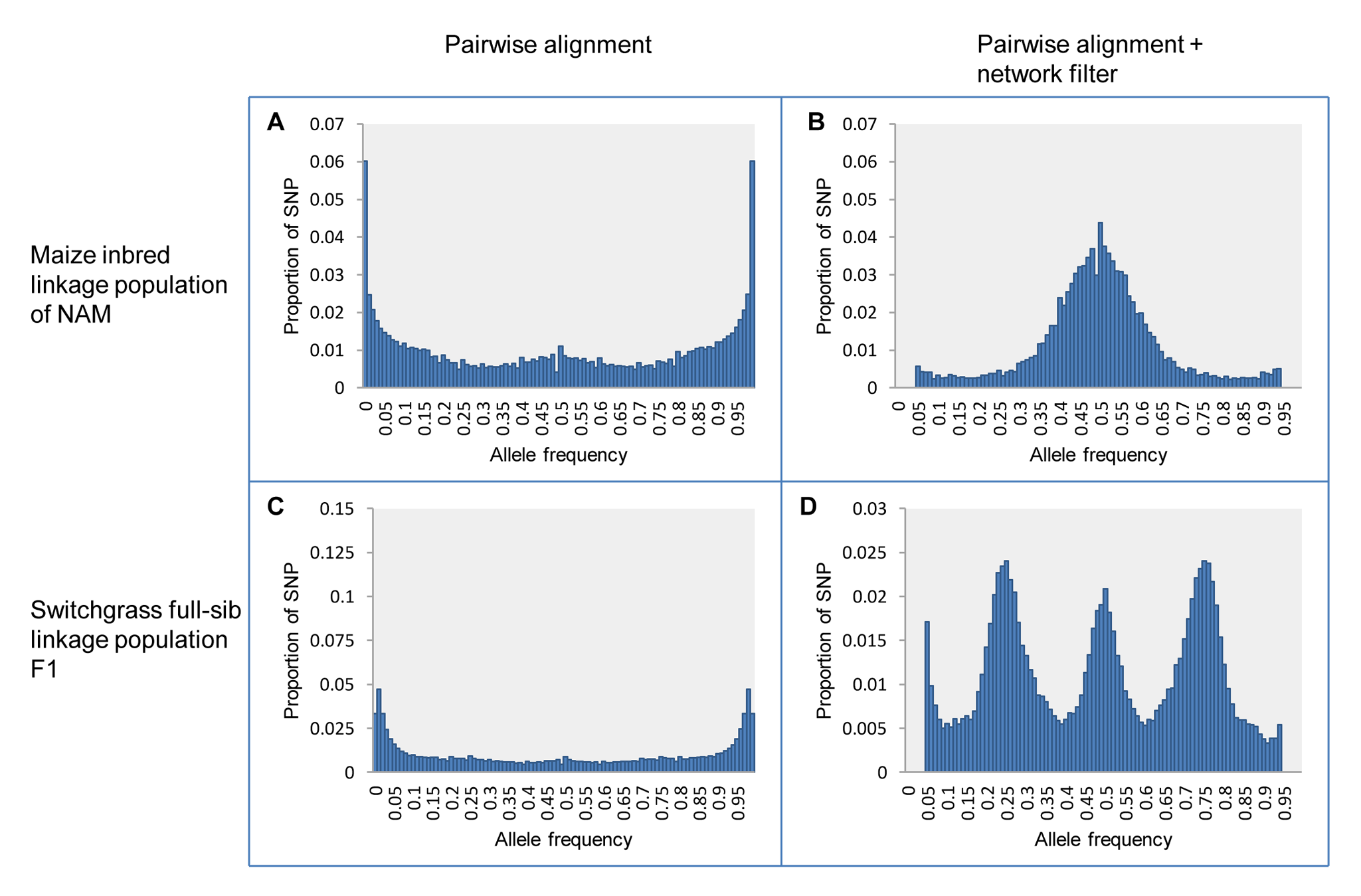

Supplement: Figure S2 — Effect of the network filter on actual allele frequency distributions in biparental populations. SNPs were called in a single family (B73×B97) from the maize NAM population [22] (A and B) and in a switchgrass full-sib F1 linkage population (C and D). We called SNPs based on finding tag pairs mismatching at a single base (A and C) and then filtered these SNPs with the network filter (B and D). The peaks at the two ends of the distributions correspond to artifactual SNPs with low minor allele frequencies resulting from sequencing errors. (TIF) [file pgen.1003215.s002.tif]

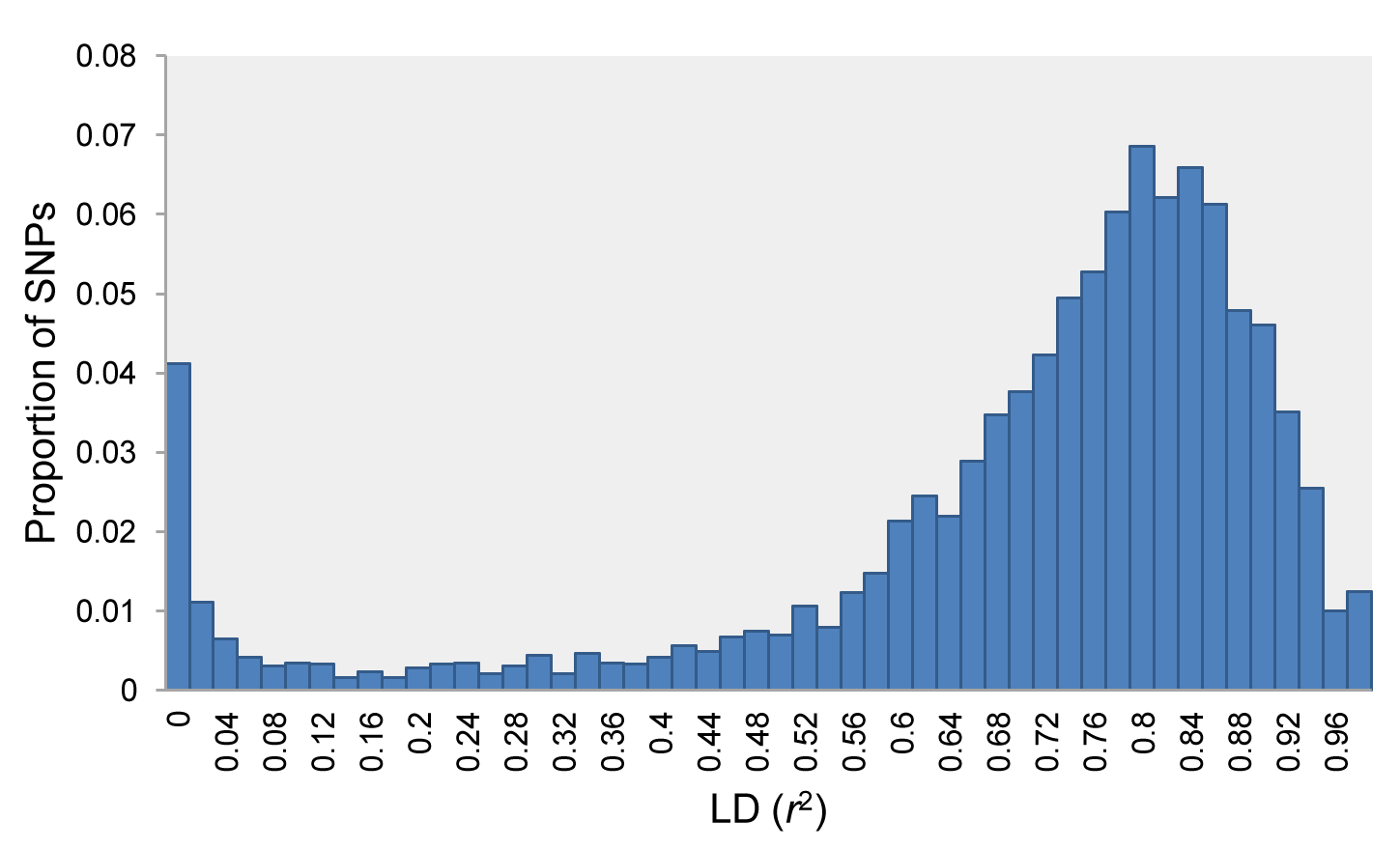

Supplement: Figure S3 — LD distribution of SNPs generated from UNEAK versus 1106 external SNP markers in the maize NAM family B73×B97 [22]. For each UNEAK SNP, the average LD (r 2) with 4 adjacent, external SNPs was calculated. (TIF) [file pgen.1003215.s003.tif]

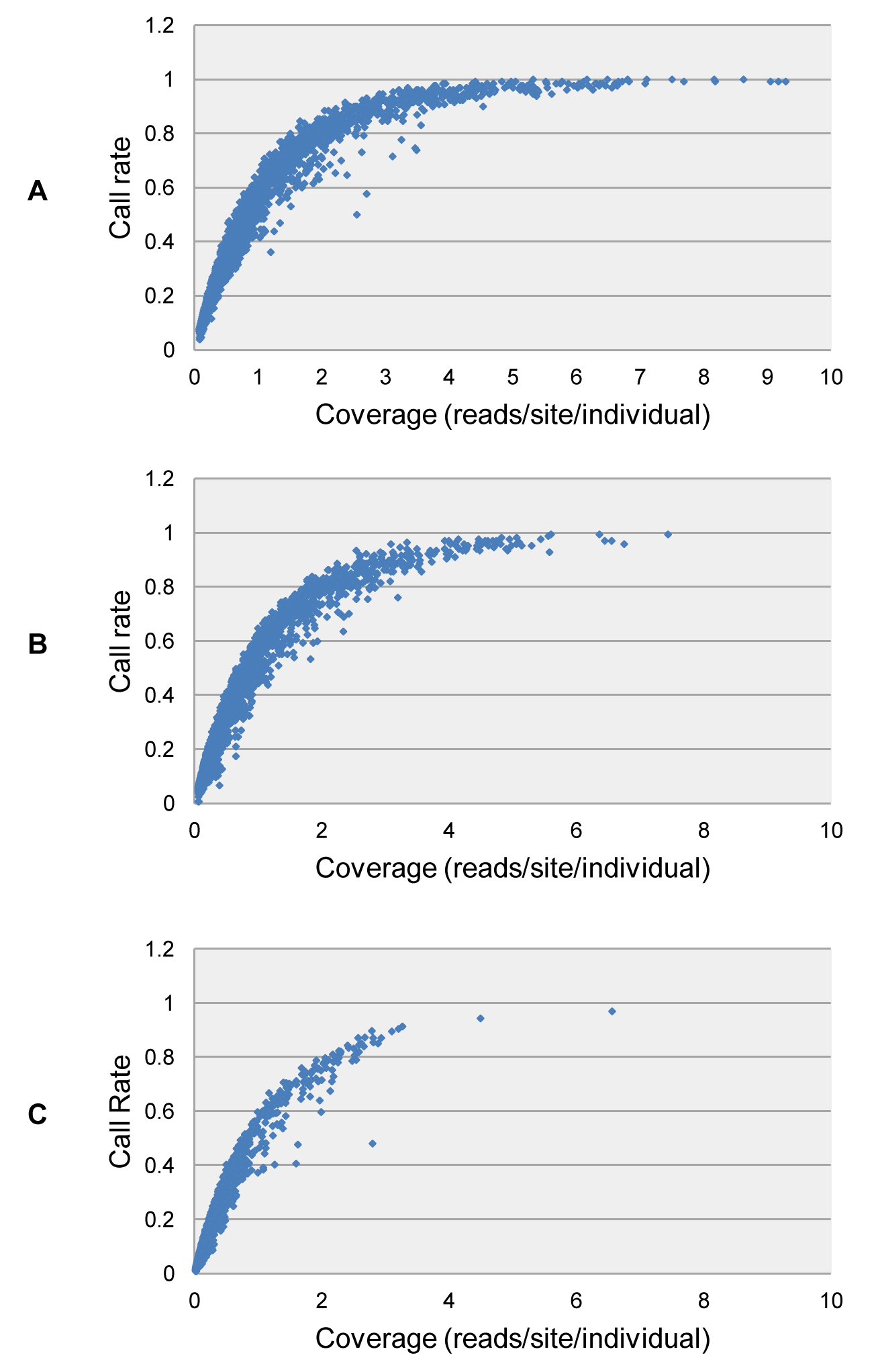

Supplement: Figure S4 — The relationship between coverage and SNP call rate in the switchgrass data sets. The call rate represents the proportion of individuals that was covered by at least one read. A total of 3,000 SNPs are plotted in each subfigure. (A) is from the full-sib population (130 individuals). (B) is from the half-sib population (168 individuals). (C) is from the association populations (540 individuals). (TIF) [file pgen.1003215.s004.tif]

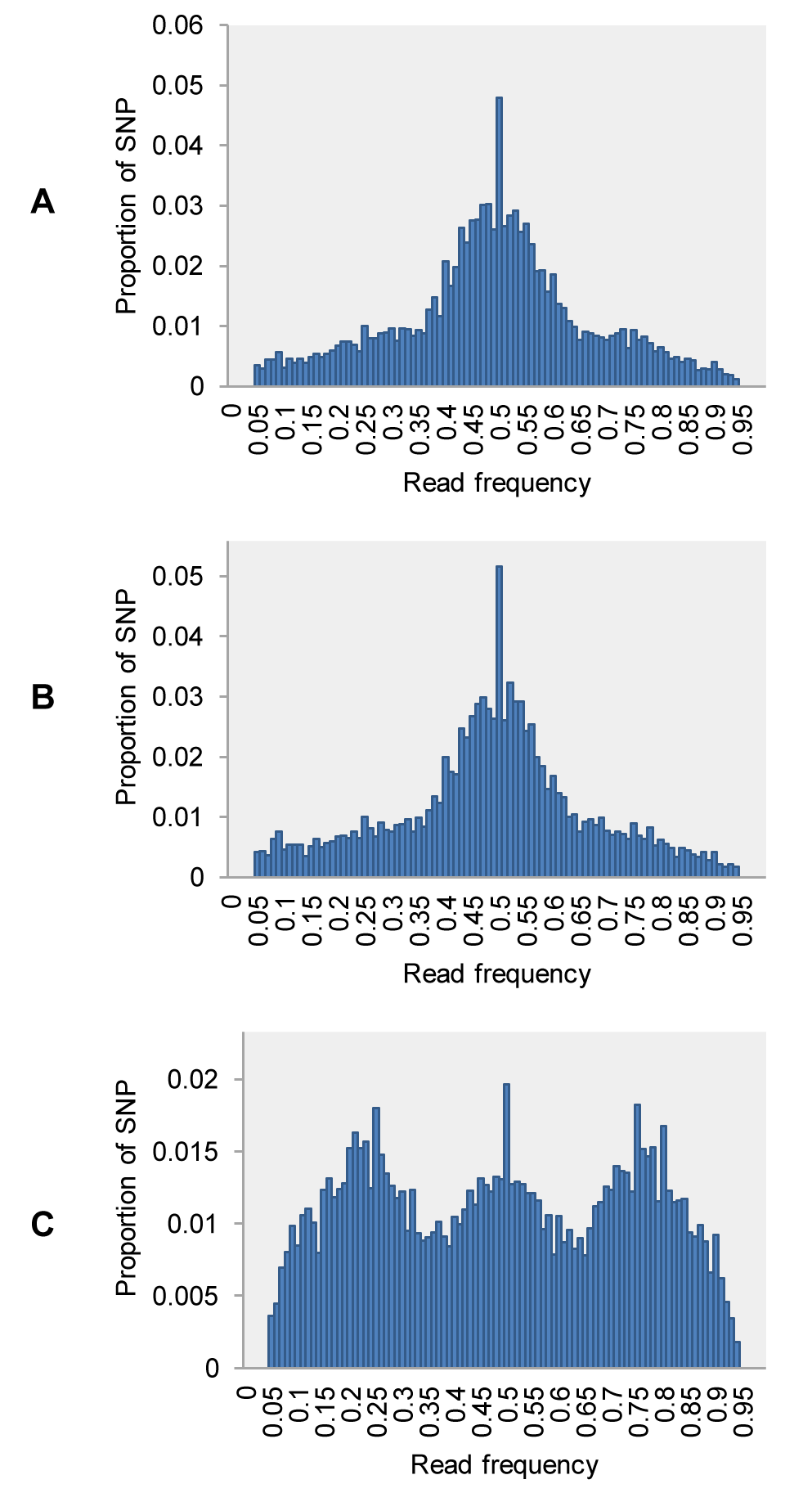

Supplement: Figure S5 — Relative depth of coverage (read frequency) of the two SNP alleles at heterozygous loci in the tetraploid U518 (A), the tetraploid U418 (B), and the octoploid K101 (C). (TIF) [file pgen.1003215.s005.tif]

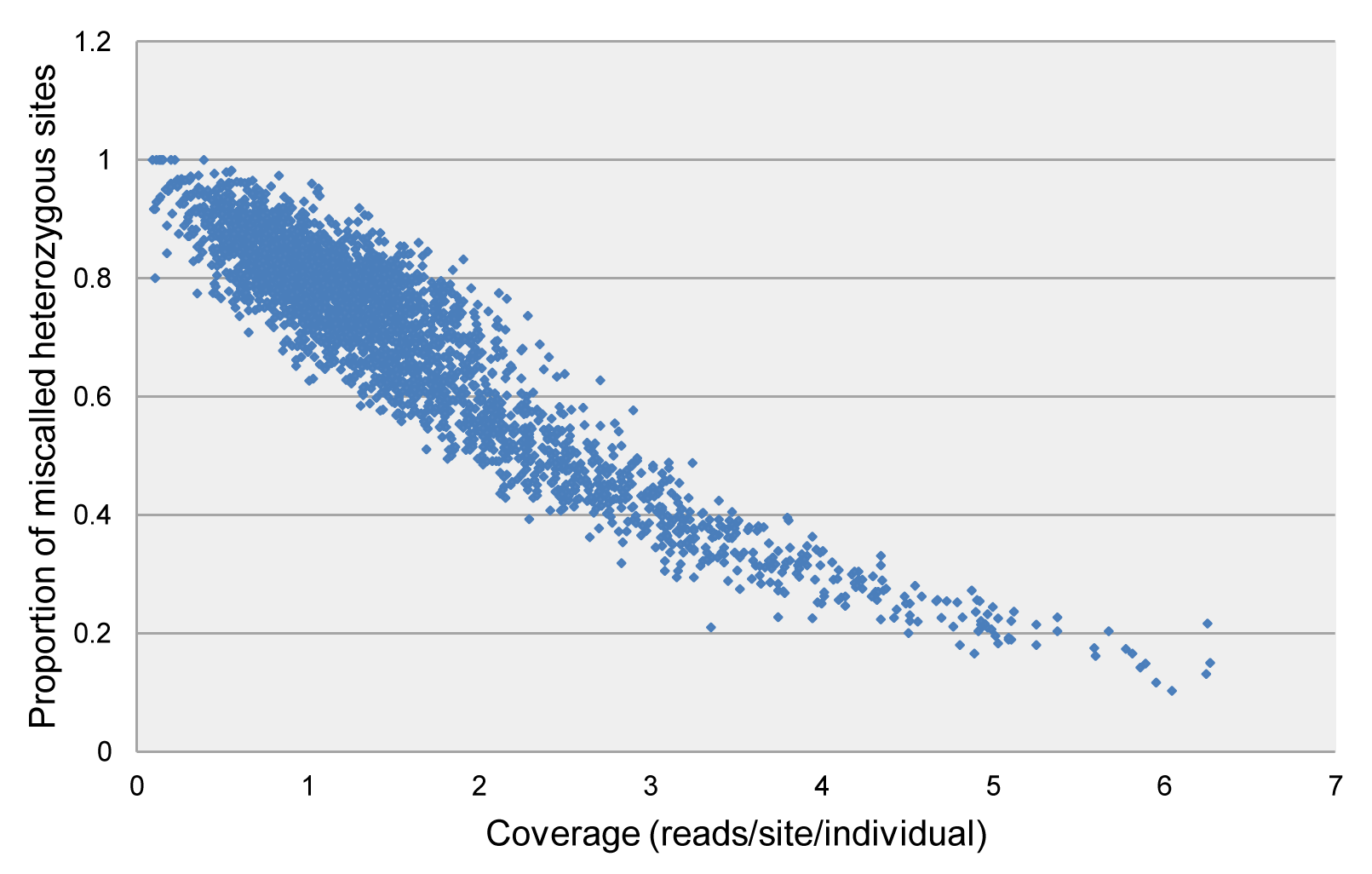

Supplement: Figure S6 — The relationship between sequencing coverage and the proportion of miscalled heterozygous genotypes. A total of 3,000 SNPs are plotted. Due to the limited sequencing depth, heterozygotes are often miscalled as homozygotes. (TIF) [file pgen.1003215.s006.tif]

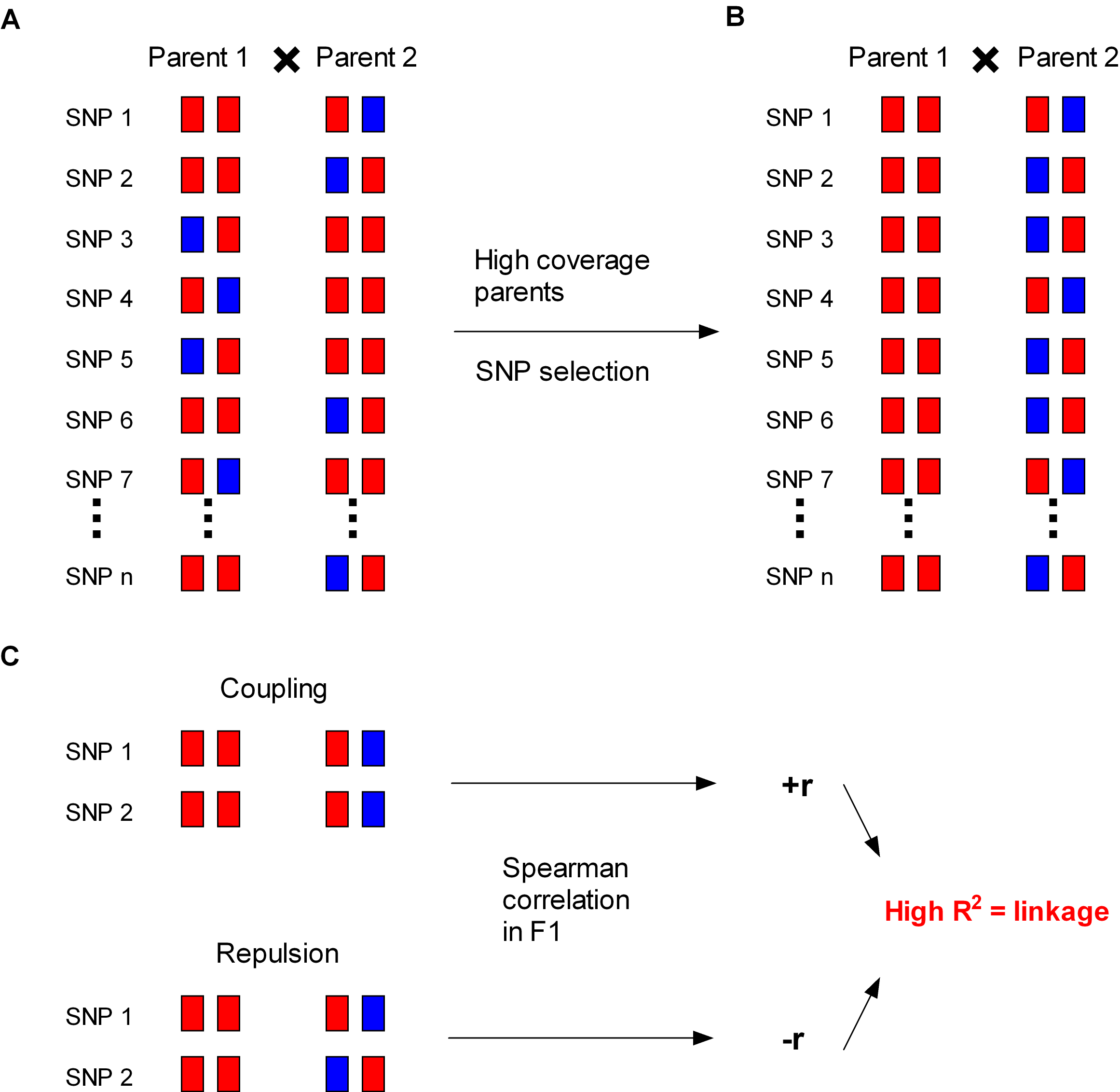

Supplement: Figure S7 — Marker selection and linkage detection via a pseudo-testcross strategy. The red and blue blocks represent bi-allelic SNPs in each site. (A) Only SNPs with allele frequencies falling within 0.2<MAF<0.3 in the F1 were selected. These SNPs must be homozygous in one parent and heterozygous in the other parent. (B) A subset of SNPs which are all homozygous in one parent and all heterozygous in the other parent, but with unknown linkage phase, were selected. (C) Close linkage of SNPs was detected based upon Spearman's rank correlation. When two SNPs are in coupling phase, the correlation is positive; when they are in repulsion, the correlation is negative. High r2 indicates tight linkage. (TIF) [file pgen.1003215.s007.tif]

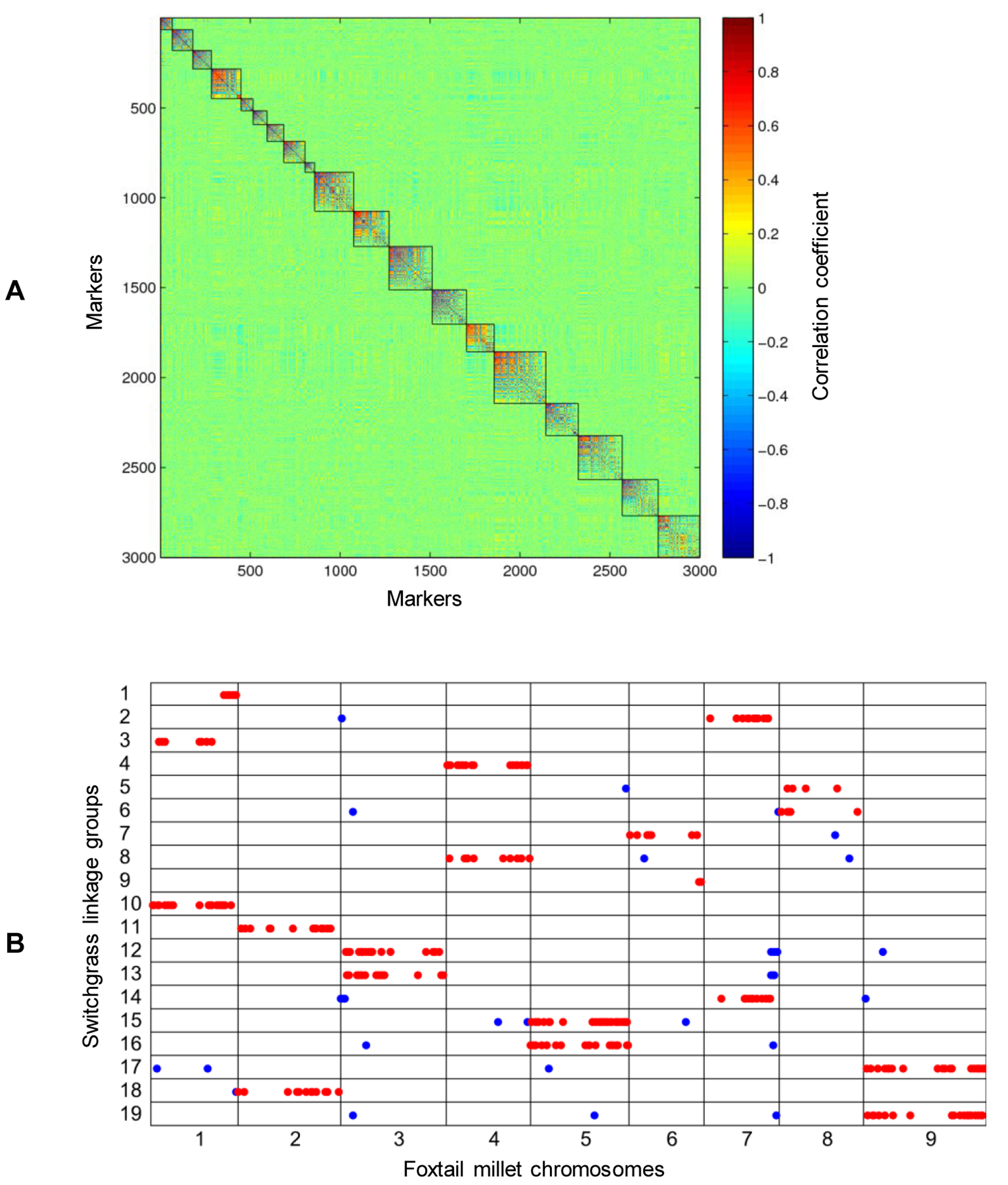

Supplement: Figure S8 — Maternal linkage groups in a biparental switchgrass family and their alignment to foxtail millet genomes. (A) 3,000 markers were clustered into 19 groups. (B) The 19 groups were aligned to the foxtail millet genome. The majority of the SNPs on each linkage group aligned to the same chromosome of the foxtail millet genome (red dots); there were some exceptions, however (blue dots). In most cases, each foxtail millet chromosome matches two linkage groups of switchgrass. The sole exception, chromosome 1, has three matching linkage groups (1, 3 and 10). Based on later analyses, we merged linkage groups 1 and 3. (TIF) [file pgen.1003215.s008.tif]

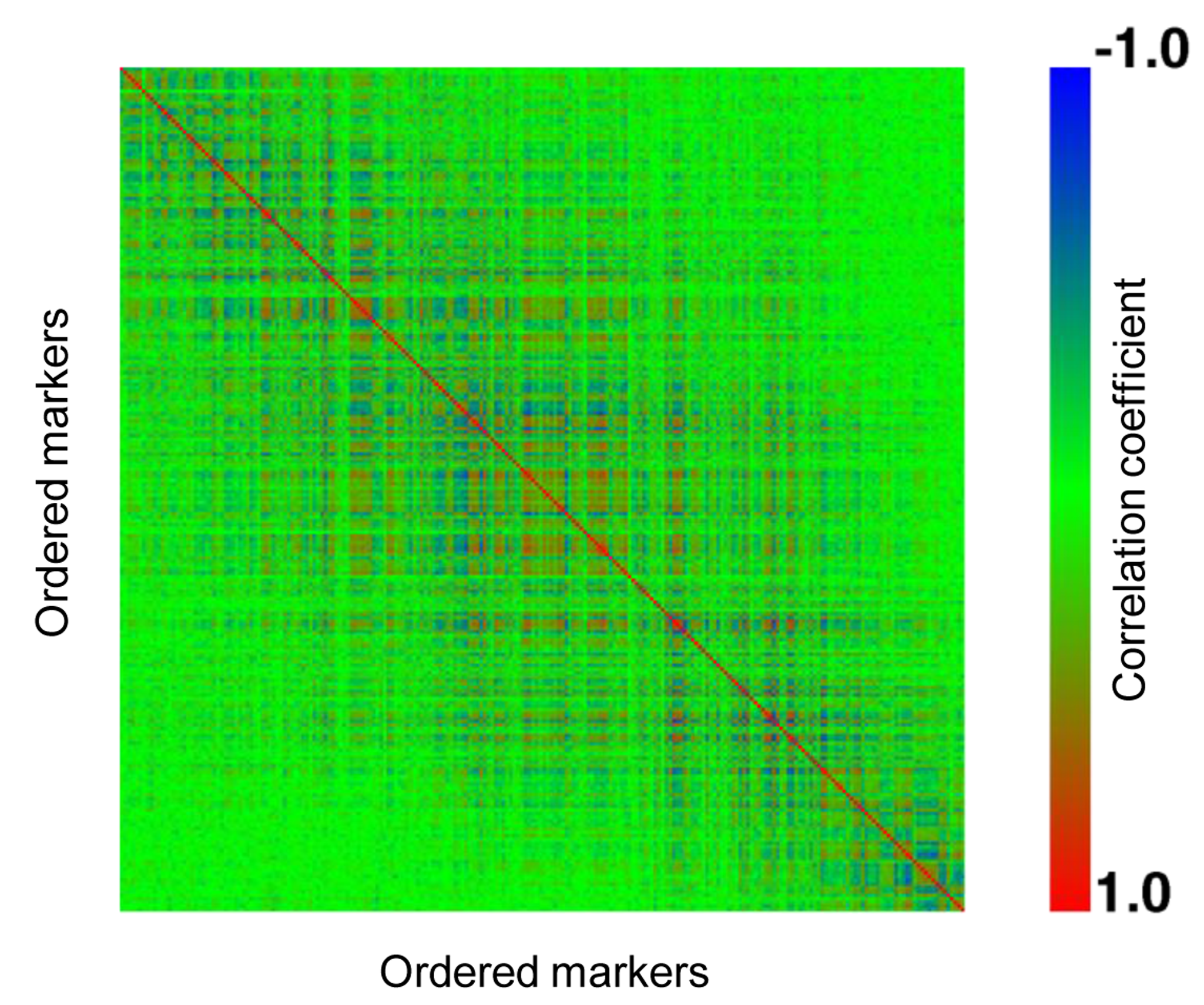

Supplement: Figure S9 — Pairwise Spearman's rank correlation coefficient (r) of ordered markers on paternal linkage group 12. A total of 228 markers were ordered based on their alignment to the foxtail millet genome. Pairwise r was calculated for these markers. The high r values were distributed along the diagonal. (TIF) [file pgen.1003215.s009.tif]

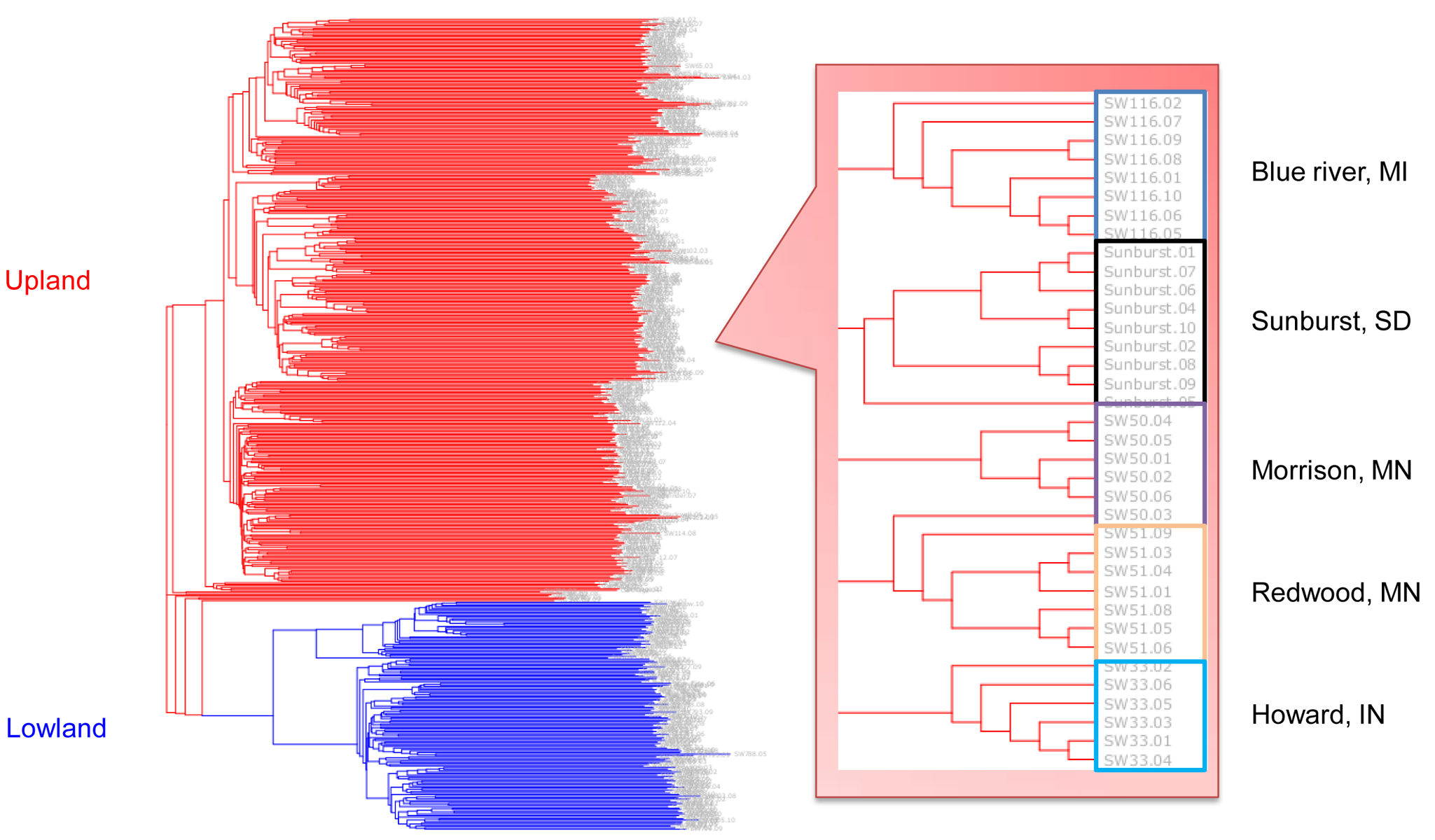

Supplement: Figure S10 — Neighbor-Joining tree of 540 individuals from 66 diverse populations of switchgrass. In the tree on the left, the red branches indicate the upland ecotype of switchgrass. The blue branches are the lowland ecotype. Details of a portion of the tree are shown on the right. Individuals within a box are from the same population. (TIF) [file pgen.1003215.s010.tif]

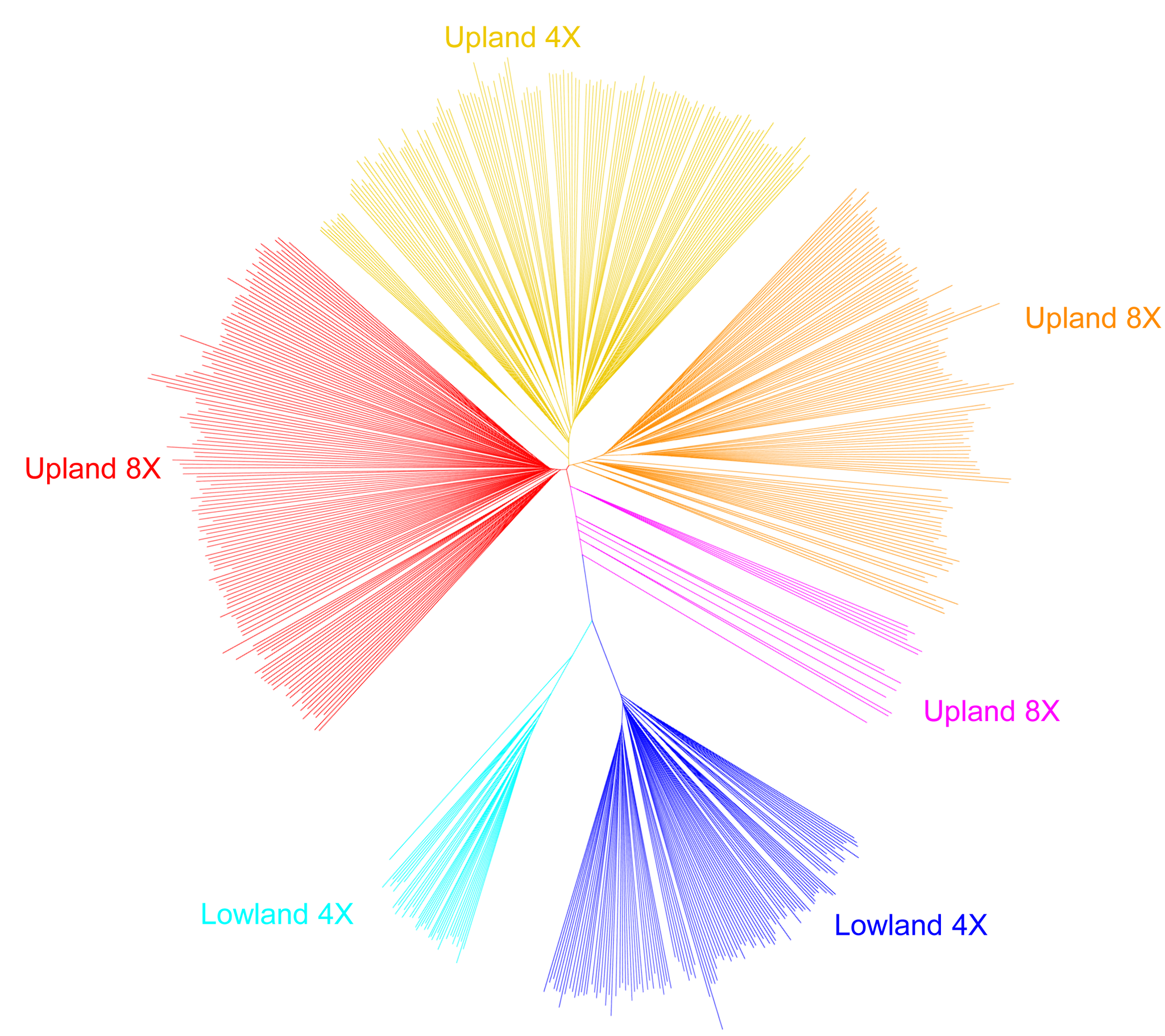

Supplement: Figure S11 — Switchgrass ecotypes with different ploidy levels resolve into distinct phylogenetic clades. (TIF) [file pgen.1003215.s011.tif]

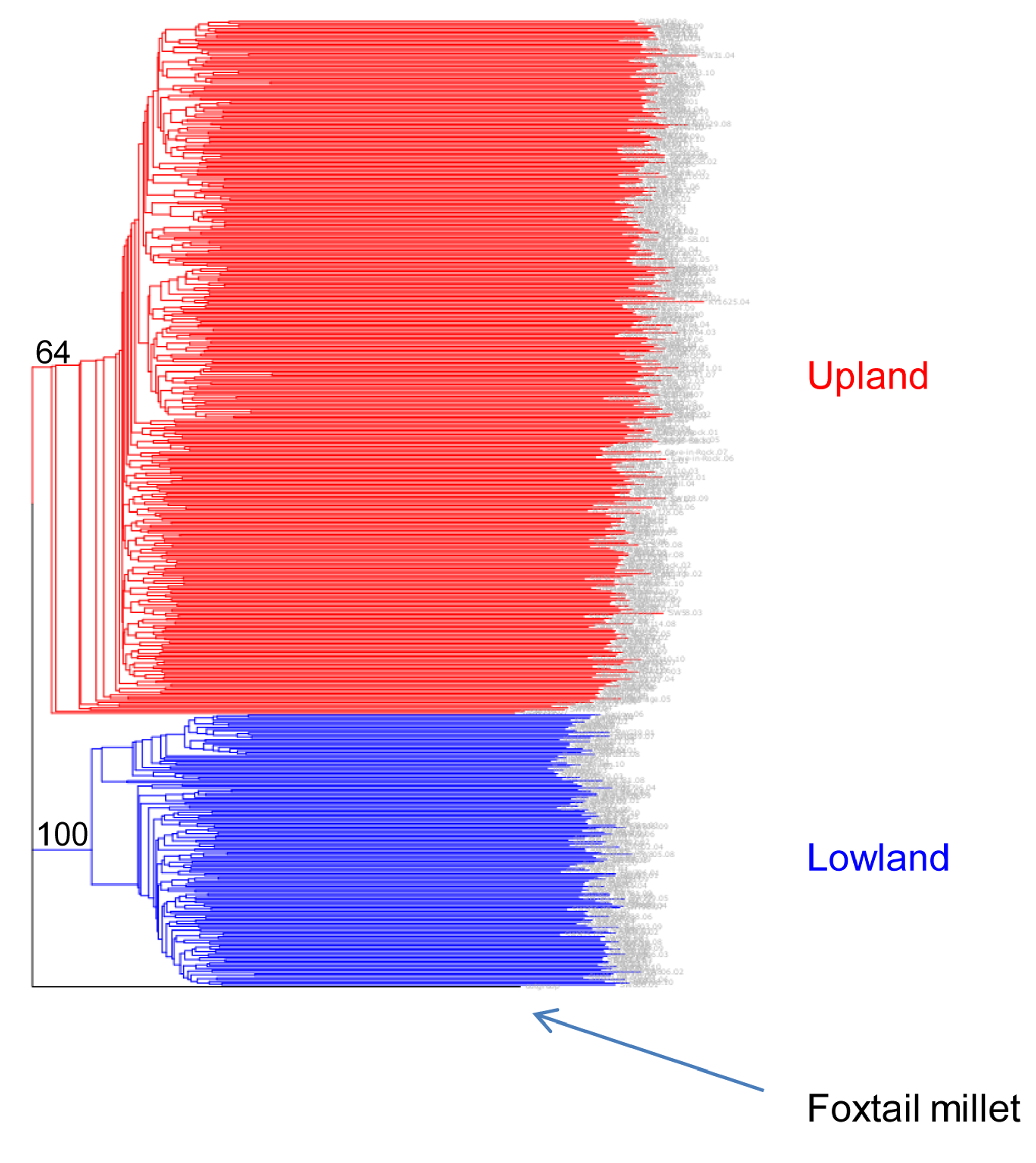

Supplement: Figure S12 — Switchgrass Neighbor-Joining phylogeny constructed with 3,144 markers. Foxtail millet was used as an outgroup. (TIF) [file pgen.1003215.s012.tif]

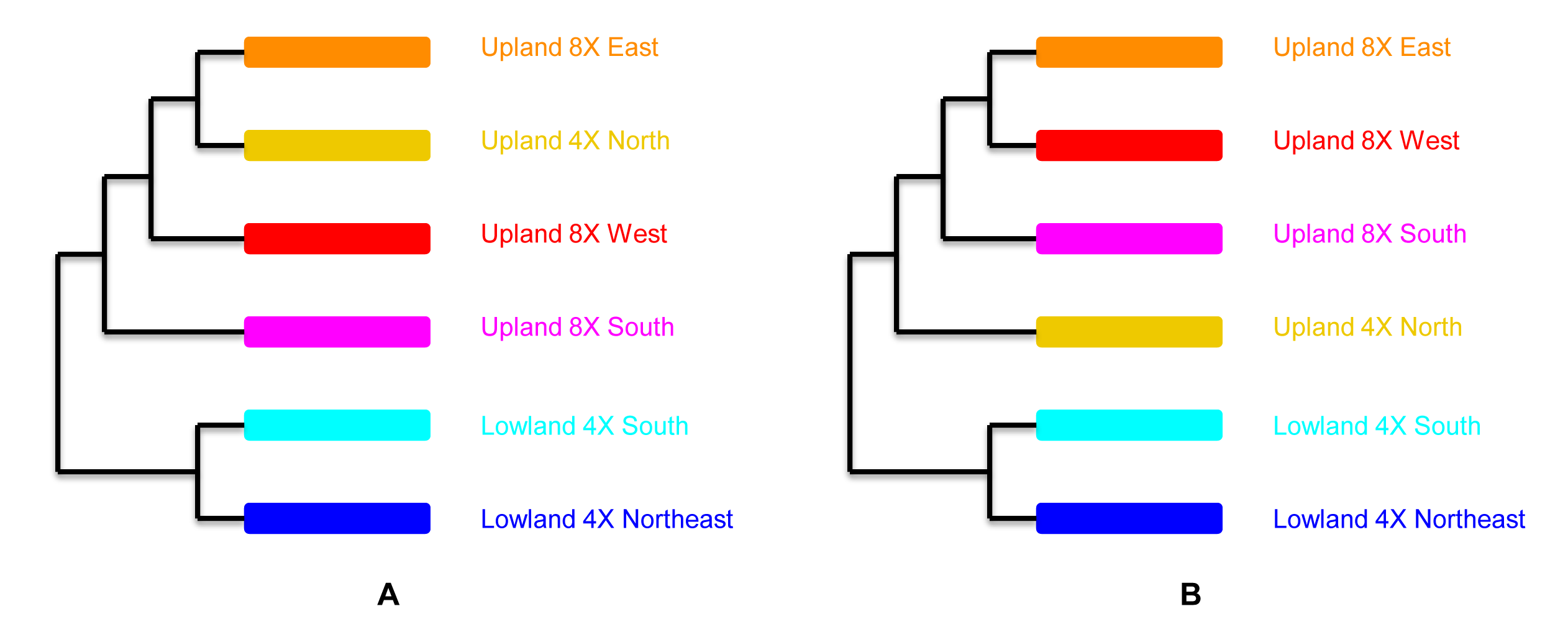

Supplement: Figure S13 — Two competing evolutionary models of upland 4× switchgrass. (A) Upland 4× arose from upland 8× switchgrass. (B) Upland 4× arose from lowland 4× switchgrass. (TIF) [file pgen.1003215.s013.tif]

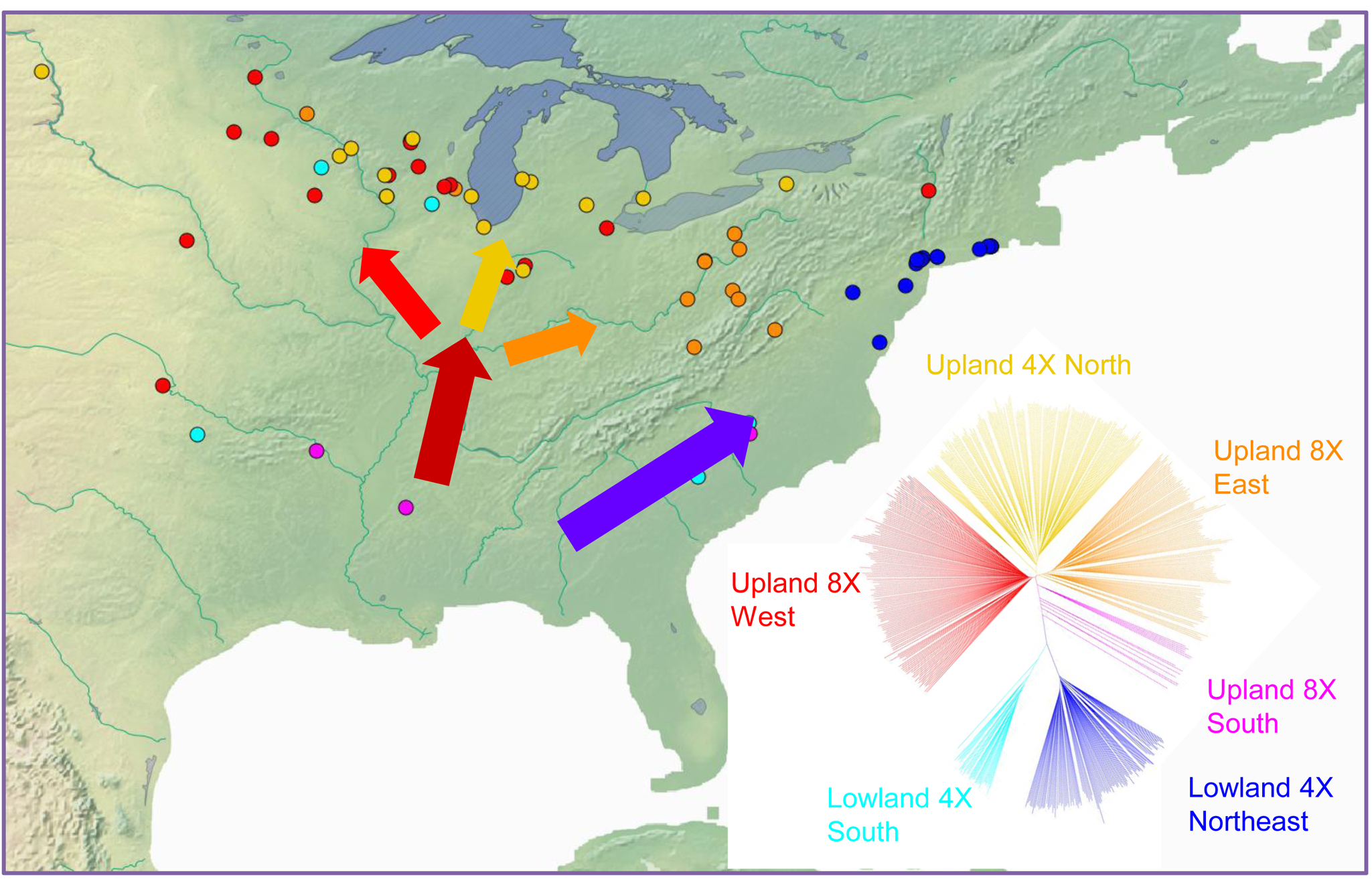

Supplement: Figure S14 — Migration patterns of switchgrass. (TIF) [file pgen.1003215.s014.tif]
