# Supplementary material for: Switchgrass Genomic Diversity, Ploidy, and Evolution: Novel Insights from a Network-Based SNP Discovery Protocol
Source: PLoS Genet. 2013 Jan 17;9(1):e1003215. doi: 10.1371/journal.pgen.1003215 (PMC3547862; doi:10.1371/journal.pgen.1003215)
Supplement: Table S1 — Background information on the germplasm used in study. (PDF) [file pgen.1003215.s015.pdf]

Table S1. Background information for germplasm used in study.

Population	No.Clones	Pedigree	Location Name	State	City*	Latitude	Longitude	Ecotype	Geo	Ploidy	Ploidy(n)
SW31	9	NaturalPopulation	Tipton	IN	Scircleville	40.30	-86.22	Upland	North	4	1
SW33	6	NaturalPopulation	Howard	IN	Howard	40.45	-86.18	Upland	West	8	1
SW38	5	NaturalPopulation	Montgomery	IN	Darlington	40.10	-86.72	Upland	West	8	2
SW40	9	NaturalPopulation	Lake	IN	East Chicago	41.63	-87.43	Upland	North	4	1
SW43	8	NaturalPopulation	Jackson	MI	Jackson	42.30	-84.28	Upland	North	4	1
SW46	9	NaturalPopulation	Hansens Island	MI	Hansens Island	42.50	-82.57	Upland	North	4	1
SW49	7	NaturalPopulation	Fillmore	MN	Peterson	43.80	-91.83	Upland	North	4	1
SW50	6	NaturalPopulation	Morrison	MN	Cushing	46.20	-94.42	Upland	West	8	1
SW51	7	NaturalPopulation	Redwood	MN	Redwood Falls	44.53	-95.08	Upland	West	8	1
SW58	10	NaturalPopulation	LeSeuer	MN	St. Peter	44.32	-93.93	Upland	West	8	3
SW63	7	NaturalPopulation	Genesee	NY	Batavia	42.93	-78.18	Upland	North	4	1
SW64	10	NaturalPopulation	Columbiana	OH	Wellsville	40.60	-80.67	Upland	East	8	1
SW65	9	NaturalPopulation	Jefferson	OH	Irondale	40.55	-80.67	Upland	East	8	1
SW102	10	NaturalPopulation	Brady's Bluff 1	WI	Trempeleau	44.02	-91.48	Upland	North	4	1
SW109	10	NaturalPopulation	Buena Vista	WI	Coddington	44.27	-89.67	Upland	West	8	2
SW110	10	NaturalPopulation	Apache Road	WI	Plainfield	44.20	-89.67	Upland	West	8	1
SW112	10	NaturalPopulation	Rocky Run 1	WI	Portage	43.47	-89.43	Upland	West	8	1
SW114	8	NaturalPopulation	Ipswich Prairie 1	WI	Benton	42.57	-90.40	Upland	North	8	1
SW115	6	NaturalPopulation	Ipswich Prairie 2	WI	Benton	42.57	-90.40	Upland	North	4	1
SW116	10	NaturalPopulation	Blue River 1	WI	Muscoda	43.20	-90.45	Upland	North	4	4
SW122	6	NaturalPopulation	Leaches Crossing	WI	Avoca	43.20	-90.33	Upland	West	8	2
SW123	10	NaturalPopulation	Waterford	WI	Potter Lake	42.78	-88.30	Upland	East	8	2
SW124	10	NaturalPopulation	Chiwaukee 1	WI	Kenosha	42.55	-87.80	Upland	North	4	1
SW127	9	NaturalPopulation	Hwy 59	WI	Eagle	42.90	-88.45	Upland	West	8	1
SW128	9	NaturalPopulation	Bald Bluff	WI	Palmyra	42.85	-88.63	Upland	West	8	1
SW129	10	NaturalPopulation	Prairie Nursery	WI	Coddington	44.33	-89.60	Upland	North	4	1
SW781	7	NaturalPopulation	Seaview	NY	Staten Island (NYC)	40.60	-74.13	Lowland	Northeast	4	3
SW782	8	NaturalPopulation	Shenandoah NP	VA	Jollett	38.48	-78.52	Upland	East	8	1
High Tide	10	NaturalTrackCultivar	Susquehanna SP	MD	Darlington	39.61	-76.15	Lowland	Northeast	4,8	1,1
Timber	8	MultisiteSynthetic	NCSU Breeding	NC	1*			Lowland	South	4	1
SW786	8	NaturalPopulation	Grand River	MI	Allendale	43.00	-86.00	Upland	North	4	1
SW787	10	NaturalPopulation	Grand Haven	MI	Grand Haven	43.09	-86.25	Upland	North	4	1
SW788	10	NaturalPopulation	Brooklyn	NY	Brooklyn (NYC)	40.68	-74.01	Lowland	Northeast	4	2
SW789	8	MultisiteSynthetic	MS SG Cycle 0	MS	2*			Low/Up	South	4,8	1,7
SW790	6	BredCultivar	MS SG Cycle 5	MS	Mississippi State	34.13	-89.03	Lowland	South	4	6
SW793	7	NaturalPopulation	Mt. Loretto (main)	NY	Tottenville	40.50	-74.22	Lowland	Northeast	4	2
SW795	8	NaturalPopulation	Elbs Pond	NY	Staten Island (NYC)	40.61	-74.08	Lowland	Northeast	4	2
SW796	10	NaturalPopulation	Gulfport	NY	Gulfport	40.62	-74.18	Lowland	Northeast	4	1
SW797	8	NaturalPopulation	NCC Campus PV#1	NY	Garden City	40.72	-73.58	Lowland	Northeast	4	1
SW798	5	NaturalPopulation	TRC Park PV#2	NY	Montauk	41.04	-71.93	Lowland	Northeast	4	1
SW799	7	NaturalPopulation	TRC Park PV#3	NY	Montauk	41.04	-71.93	Lowland	Northeast	4	1
SW802	6	NaturalPopulation	NCC Campus PV#6	NY	Garden City	40.72	-73.58	Lowland	Northeast	4	2
SW803	9	NaturalPopulation	TRC Park PV#7	NY	Montauk	41.04	-71.93	Lowland	Northeast	4	1
SW805	10	NaturalPopulation	Hither Hills SP	NY	Montauk	41.02	-72.01	Lowland	Northeast	4	1
SW806	9	NaturalPopulation	Sammy's Beach	NY	Bridgehampton	40.94	-72.28	Lowland	Northeast	4	1
SW808	10	NaturalPopulation	Morgantown	WV	Morgantown	39.68	-79.81	Upland	East	8	1
SW809	10	NaturalPopulation	Camp Dawson	WV	Tunnelton	39.41	-79.66	Upland	East	8	1
Blackwell	10	NaturalTrackCultivar	Blackwell	OK	Blackwell	36.76	-97.24	Upland	West	8	1
Carthage	6	NaturalTrackCultivar	Carthage	NC	Carthage	35.31	-79.30	Upland	South	8	2
Cave-in-Rock	10	NaturalTrackCultivar	Cave-in-Rock	IL	Cave-in-Rock	37.47	-88.16	Upland	East	8	1
Dacotah	8	NaturalTrackCultivar	Dacotah	ND	Raleigh	46.38	-100.94	Upland	North	4	1
Kanlow	9	NaturalTrackCultivar	Kanlow	OK	Wetumka	35.26	-96.18	Lowland	South	4	1
KY1625	10	NaturalTrackCultivar	KY1625	WV	Thurmond	37.94	-80.99	Upland	East	8	1
Pathfinder	10	BredCultivar	Pathfinder	NE	Mead	41.20	-96.50	Upland	West	8	2
Shelter	9	NaturalTrackCultivar	Shelter	WV	St. Marys	39.41	-81.20	Upland	East	8	1
Sunburst	9	BredCultivar	Sunburst	SD	Jefferson	42.60	-92.60	Upland	West	8	1
SWG32	7	NaturalPopulation	Harlem Hills Prairie	IL	Love's Park	42.33	-89.02	Lowland	South	4	1
SWG39	8	NaturalPopulation	Hayden Prairie	IA	Chester	43.44	-92.38	Lowland	South	4	3
WS4U	8	MultisiteSynthetic	WS4U	WI	3*			Upland	North	4	1
WS98-SB	8	NaturalPopulation	Sterling Barrens	WI	Cushing	45.08	-92.83	Upland	East/North	4,6,8	4,1,3
ECS-1	5	NaturalPopulation	Route 72/563	NJ	Chatsworth	39.82	-74.53	Lowland	Notheast	4	1
ECS-2	4	NaturalPopulation	Toledo	OH	Toledo	41.58	-83.67	Upland	West	8	1
ECS-10	8	NaturalPopulation	Allegheny River	PA	East Brady	40.95	-79.62	Upland	East	8	1
ECS-11	5	NaturalPopulation	Franklin	PA	Franklin	41.40	-79.77	Upland	East	8	1
ECS-12	7	NaturalPopulation	Albany	NY	Colonie	42.72	-73.83	Upland	West	8	1
ECS-6	3	NaturalPopulation	Truitt's Landing	MD	Girdletree	38.08	-75.33	Lowland	Northeast	4	1

All germplasm originates from locations in the United States of America. City indicates the closest mapable city to the collection location. In three cases (indicated by *), there is no single GPS location, the region of collection is too large: 1=North Carolina, South Carolina, Georgia ; 2=Arkansas and Mississippi ; 3=North Central USA . "Geo" is the geographic subgroup. "Ploidy" indicates the level , i.e., 8=8X=octoploid) . " Ploidy (n)" = the number of clones in the population where ploidy was estimated, using flow cytometry. For a description of the flow cytometry methods, see [2].
