# Supplementary material for: Switchgrass Genomic Diversity, Ploidy, and Evolution: Novel Insights from a Network-Based SNP Discovery Protocol
Source: PLoS Genet. 2013 Jan 17;9(1):e1003215. doi: 10.1371/journal.pgen.1003215 (PMC3547862; doi:10.1371/journal.pgen.1003215)
Supplement: Table S2 — Individual clones sequenced in the association panel. (PDF) [file pgen.1003215.s016.pdf]

Table S2. Indivdidual clones sequenced in the association panel

Individual	Ecotype	Ploidy*
Blackwell.01	Upland	Na
Blackwell.02	Upland	Na
Blackwell.03	Upland	Na
Blackwell.04	Upland	8
Blackwell.05	Upland	Na
Blackwell.06	Upland	Na
Blackwell.07	Upland	Na
Blackwell.08	Upland	Na
Blackwell.09	Upland	Na
Blackwell.10	Upland	Na
Carthage.01	Upland	Na
Carthage.02	Upland	Na
Carthage.03	Upland	8
Carthage.04	Upland	8
Carthage.05	Upland	Na
Carthage.06	Upland	Na
Cave-in-Rock.01	Upland	Na
Cave-in-Rock.02	Upland	Na
Cave-in-Rock.03	Upland	Na
Cave-in-Rock.04	Upland	Na
Cave-in-Rock.05	Upland	Na
Cave-in-Rock.06	Upland	Na
Cave-in-Rock.07	Upland	Na
Cave-in-Rock.08	Upland	8
Cave-in-Rock.09	Upland	Na
Cave-in-Rock.10	Upland	Na
Dacotah.01	Upland	Na
Dacotah.02	Upland	Na
Dacotah.03	Upland	Na
Dacotah.04	Upland	Na
Dacotah.05	Upland	Na
Dacotah.06	Upland	Na
Dacotah.07	Upland	Na
Dacotah.08	Upland	4
ECS-1.02	Lowland	Na
ECS-1.03	Lowland	Na
ECS-1.05	Lowland	Na
ECS-1.06	Lowland	4
ECS-1.07	Lowland	Na
ECS-10.01	Upland	Na
ECS-10.03	Upland	Na
ECS-10.04	Upland	Na
ECS-10.05	Upland	Na
ECS-10.07	Upland	8
ECS-10.08	Upland	Na

ECS-10.09	Upland	Na
ECS-10.10	Upland	Na
ECS-11.01	Upland	Na
ECS-11.02	Upland	Na
ECS-11.04	Upland	Na
ECS-11.05	Upland	Na
ECS-11.07	Upland	8
ECS-12.01	Upland	Na
ECS-12.02	Upland	Na
ECS-12.03	Upland	Na
ECS-12.04	Upland	8
ECS-12.05	Upland	8
ECS-12.06	Upland	Na
ECS-12.07	Upland	Na
ECS-2.01	Upland	Na
ECS-2.02	Upland	Na
ECS-2.03	Upland	Na
ECS-2.04	Upland	8
ECS-2.06	Upland	Na
ECS-2.07	Upland	Na
ECS-6.01	Lowland	Na
ECS-6.02	Lowland	Na
ECS-6.03	Lowland	4
High-Tide.01	Lowland	Na
High-Tide.02	Lowland	Na
High-Tide.03	Lowland	Na
High-Tide.04	Lowland	Na
High-Tide.05	Lowland	Na
High-Tide.06	Lowland	Na
High-Tide.07	Lowland	8
High-Tide.08	Lowland	Na
High-Tide.09	Lowland	Na
High-Tide.10	Lowland	4
Kanlow.01	Lowland	Na
Kanlow.02	Lowland	Na
Kanlow.04	Lowland	Na
Kanlow.05	Lowland	Na
Kanlow.06	Lowland	Na
Kanlow.07	Lowland	Na
Kanlow.08	Lowland	Na
Kanlow.09	Lowland	Na
Kanlow.10	Lowland	4
KY1625.01	Upland	Na
KY1625.02	Upland	Na
KY1625.03	Upland	Na
KY1625.04	Upland	Na
KY1625.05	Upland	Na

KY1625.06	Upland	Na
KY1625.07	Upland	Na
KY1625.08	Upland	Na
KY1625.09	Upland	Na
KY1625.10	Upland	8
Pathfinder.01	Upland	Na
Pathfinder.02	Upland	Na
Pathfinder.03	Upland	Na
Pathfinder.04	Upland	Na
Pathfinder.05	Upland	Na
Pathfinder.06	Upland	Na
Pathfinder.07	Upland	Na
Pathfinder.08	Upland	8
Pathfinder.09	Upland	Na
Pathfinder.10	Upland	8
Shelter.01	Upland	Na
Shelter.02	Upland	Na
Shelter.04	Upland	Na
Shelter.05	Upland	Na
Shelter.06	Upland	Na
Shelter.07	Upland	Na
Shelter.08	Upland	Na
Shelter.09	Upland	Na
Shelter.10	Upland	8
Sunburst.01	Upland	Na
Sunburst.02	Upland	Na
Sunburst.04	Upland	Na
Sunburst.05	Upland	Na
Sunburst.06	Upland	Na
Sunburst.07	Upland	Na
Sunburst.08	Upland	Na
Sunburst.09	Upland	Na
Sunburst.10	Upland	8
SW102.01	Upland	Na
SW102.02	Upland	Na
SW102.03	Upland	4
SW102.04	Upland	Na
SW102.05	Upland	Na
SW102.06	Upland	Na
SW102.07	Upland	Na
SW102.08	Upland	Na
SW102.09	Upland	Na
SW102.10	Upland	Na
SW109.01	Upland	Na
SW109.02	Upland	Na
SW109.03	Upland	8
SW109.04	Upland	Na

SW109.05	Upland	Na
SW109.06	Upland	Na
SW109.07	Upland	Na
SW109.08	Upland	Na
SW109.09	Upland	Na
SW109.10	Upland	8
SW110.01	Upland	Na
SW110.02	Upland	Na
SW110.03	Upland	8
SW110.04	Upland	Na
SW110.05	Upland	Na
SW110.06	Upland	Na
SW110.07	Upland	Na
SW110.08	Upland	Na
SW110.09	Upland	Na
SW110.10	Upland	Na
SW112.01	Upland	Na
SW112.02	Upland	Na
SW112.03	Upland	8
SW112.04	Upland	Na
SW112.05	Upland	Na
SW112.06	Upland	Na
SW112.07	Upland	Na
SW112.08	Upland	Na
SW112.09	Upland	Na
SW112.10	Upland	Na
SW114.01	Upland	4
SW114.02	Upland	4
SW114.03	Upland	4
SW114.04	Upland	4
SW114.05	Upland	8
SW114.06	Upland	8
SW114.07	Upland	8
SW114.08	Upland	8
SW115.01	Upland	4
SW115.02	Upland	4
SW115.03	Upland	4
SW115.04	Upland	4
SW115.05	Upland	4
SW115.06	Upland	4
SW116.01	Upland	4
SW116.02	Upland	Na
SW116.03	Upland	Na
SW116.04	Upland	4
SW116.05	Upland	Na
SW116.06	Upland	Na
SW116.07	Upland	Na

SW116.08	Upland	4
SW116.09	Upland	4
SW116.10	Upland	Na
SW122.01	Upland	8
SW122.02	Upland	Na
SW122.03	Upland	Na
SW122.04	Upland	8
SW122.05	Upland	Na
SW122.06	Upland	Na
SW123.01	Upland	Na
SW123.02	Upland	8
SW123.03	Upland	8
SW123.04	Upland	Na
SW123.05	Upland	Na
SW123.06	Upland	Na
SW123.07	Upland	Na
SW123.08	Upland	Na
SW123.09	Upland	Na
SW123.10	Upland	Na
SW124.01	Upland	Na
SW124.02	Upland	Na
SW124.03	Upland	4
SW124.04	Upland	Na
SW124.05	Upland	Na
SW124.06	Upland	Na
SW124.07	Upland	Na
SW124.08	Upland	Na
SW124.09	Upland	Na
SW124.10	Upland	Na
SW127.01	Upland	Na
SW127.02	Upland	Na
SW127.03	Upland	8
SW127.04	Upland	Na
SW127.05	Upland	Na
SW127.06	Upland	Na
SW127.07	Upland	Na
SW127.08	Upland	Na
SW127.09	Upland	Na
SW128.01	Upland	Na
SW128.02	Upland	Na
SW128.03	Upland	Na
SW128.04	Upland	8
SW128.05	Upland	Na
SW128.06	Upland	Na
SW128.07	Upland	Na
SW128.08	Upland	Na
SW128.10	Upland	Na

SW129.01	Upland	Na
SW129.02	Upland	Na
SW129.03	Upland	Na
SW129.04	Upland	4
SW129.05	Upland	Na
SW129.06	Upland	Na
SW129.07	Upland	Na
SW129.08	Upland	Na
SW129.09	Upland	Na
SW129.10	Upland	Na
SW31.01	Upland	Na
SW31.02	Upland	4
SW31.03	Upland	Na
SW31.04	Upland	Na
SW31.05	Upland	Na
SW31.06	Upland	Na
SW31.07	Upland	Na
SW31.08	Upland	Na
SW31.09	Upland	Na
SW33.01	Upland	8
SW33.02	Upland	Na
SW33.03	Upland	Na
SW33.04	Upland	Na
SW33.05	Upland	Na
SW33.06	Upland	Na
SW38.02	Upland	Na
SW38.03	Upland	Na
SW38.04	Upland	8
SW38.05	Upland	8
SW38.08	Upland	Na
SW40.01	Upland	Na
SW40.02	Upland	Na
SW40.03	Upland	Na
SW40.05	Upland	Na
SW40.06	Upland	Na
SW40.07	Upland	Na
SW40.08	Upland	Na
SW40.09	Upland	4
SW40.10	Upland	Na
SW43.02	Upland	Na
SW43.04	Upland	4
SW43.05	Upland	Na
SW43.06	Upland	Na
SW43.07	Upland	Na
SW43.08	Upland	Na
SW43.09	Upland	Na
SW43.10	Upland	Na

SW46.01	Upland	Na
SW46.02	Upland	Na
SW46.03	Upland	Na
SW46.04	Upland	4
SW46.05	Upland	Na
SW46.06	Upland	Na
SW46.08	Upland	Na
SW46.09	Upland	Na
SW46.10	Upland	Na
SW49.01	Upland	Na
SW49.02	Upland	Na
SW49.03	Upland	Na
SW49.04	Upland	4
SW49.05	Upland	Na
SW49.06	Upland	Na
SW49.07	Upland	Na
SW50.01	Upland	Na
SW50.02	Upland	8
SW50.03	Upland	Na
SW50.04	Upland	Na
SW50.05	Upland	Na
SW50.06	Upland	Na
SW51.01	Upland	8
SW51.03	Upland	Na
SW51.04	Upland	Na
SW51.05	Upland	Na
SW51.06	Upland	Na
SW51.08	Upland	Na
SW51.09	Upland	Na
SW58.01	Upland	Na
SW58.02	Upland	Na
SW58.03	Upland	Na
SW58.04	Upland	Na
SW58.05	Upland	Na
SW58.06	Upland	8
SW58.07	Upland	8
SW58.08	Upland	Na
SW58.09	Upland	8
SW58.10	Upland	Na
SW63.02	Upland	Na
SW63.03	Upland	Na
SW63.04	Upland	Na
SW63.05	Upland	Na
SW63.06	Upland	4
SW63.07	Upland	Na
SW63.08	Upland	Na
SW64.01	Upland	Na

SW64.02	Upland	Na
SW64.03	Upland	8
SW64.04	Upland	Na
SW64.05	Upland	Na
SW64.06	Upland	Na
SW64.07	Upland	Na
SW64.08	Upland	Na
SW64.09	Upland	Na
SW64.10	Upland	Na
SW65.02	Upland	8
SW65.03	Upland	8
SW65.04	Upland	8
SW65.05	Upland	8
SW65.06	Upland	8
SW65.07	Upland	8
SW65.08	Upland	8
SW65.09	Upland	4
SW65.10	Upland	4
SW781.03	Lowland	Na
SW781.04	Lowland	4
SW781.05	Lowland	Na
SW781.06	Lowland	Na
SW781.07	Lowland	4
SW781.08	Lowland	4
SW781.09	Lowland	Na
SW782.01	Upland	Na
SW782.02	Upland	Na
SW782.03	Upland	Na
SW782.06	Upland	Na
SW782.07	Upland	Na
SW782.08	Upland	Na
SW782.09	Upland	Na
SW782.10	Upland	8
SW786.01	Upland	4
SW786.02	Upland	4
SW786.03	Upland	4
SW786.04	Upland	4
SW786.06	Upland	4
SW786.07	Upland	4
SW786.09	Upland	error
SW786.10	Upland	4
SW787.01	Upland	Na
SW787.02	Upland	Na
SW787.03	Upland	Na
SW787.04	Upland	Na
SW787.05	Upland	4
SW787.06	Upland	Na

SW787.07	Upland	Na
SW787.08	Upland	Na
SW787.09	Upland	Na
SW787.10	Upland	Na
SW788.01	Lowland	Na
SW788.02	Lowland	Na
SW788.03	Lowland	Na
SW788.04	Lowland	Na
SW788.05	Lowland	4
SW788.06	Lowland	Na
SW788.07	Lowland	Na
SW788.08	Lowland	4
SW788.09	Lowland	Na
SW788.10	Lowland	Na
SW789.01	Low/Up	4
SW789.02	Low/Up	8
SW789.04	Low/Up	8
SW789.05	Low/Up	8
SW789.06	Low/Up	8
SW789.07	Low/Up	8
SW789.08	Low/Up	8
SW789.09	Low/Up	8
SW790.01	Lowland	4
SW790.02	Lowland	4
SW790.03	Lowland	4
SW790.04	Lowland	4
SW790.06	Lowland	4
SW790.07	Lowland	4
SW793.01	Lowland	4
SW793.02	Lowland	Na
SW793.03	Lowland	4
SW793.06	Lowland	Na
SW793.07	Lowland	Na
SW793.09	Lowland	Na
SW793.10	Lowland	Na
SW795.01	Lowland	Na
SW795.02	Lowland	4
SW795.04	Lowland	Na
SW795.06	Lowland	Na
SW795.07	Lowland	Na
SW795.08	Lowland	Na
SW795.09	Lowland	4
SW795.10	Lowland	Na
SW796.01	Lowland	Na
SW796.02	Lowland	Na
SW796.03	Lowland	Na
SW796.04	Lowland	Na

SW796.05	Lowland	Na
SW796.06	Lowland	Na
SW796.07	Lowland	Na
SW796.08	Lowland	Na
SW796.09	Lowland	4
SW796.10	Lowland	Na
SW797.01	Lowland	Na
SW797.02	Lowland	Na
SW797.03	Lowland	Na
SW797.04	Lowland	Na
SW797.05	Lowland	Na
SW797.06	Lowland	Na
SW797.09	Lowland	4
SW797.10	Lowland	Na
SW798.01	Lowland	4
SW798.05	Lowland	Na
SW798.06	Lowland	Na
SW798.09	Lowland	Na
SW798.10	Lowland	Na
SW799.02	Lowland	Na
SW799.03	Lowland	Na
SW799.05	Lowland	Na
SW799.07	Lowland	Na
SW799.08	Lowland	Na
SW799.09	Lowland	4
SW799.10	Lowland	Na
SW802.01	Lowland	4
SW802.02	Lowland	Na
SW802.03	Lowland	Na
SW802.04	Lowland	4
SW802.05	Lowland	Na
SW802.06	Lowland	Na
SW803.02	Lowland	Na
SW803.03	Lowland	Na
SW803.04	Lowland	Na
SW803.05	Lowland	Na
SW803.06	Lowland	Na
SW803.07	Lowland	Na
SW803.08	Lowland	4
SW803.09	Lowland	Na
SW803.10	Lowland	Na
SW805.01	Lowland	Na
SW805.02	Lowland	Na
SW805.03	Lowland	4
SW805.04	Lowland	Na
SW805.05	Lowland	Na
SW805.06	Lowland	Na

SW805.07	Lowland	Na
SW805.08	Lowland	Na
SW805.09	Lowland	Na
SW805.10	Lowland	Na
SW806.01	Lowland	Na
SW806.02	Lowland	Na
SW806.03	Lowland	4
SW806.04	Lowland	Na
SW806.05	Lowland	Na
SW806.06	Lowland	Na
SW806.07	Lowland	Na
SW806.08	Lowland	Na
SW806.09	Lowland	Na
SW808.01	Upland	Na
SW808.02	Upland	Na
SW808.03	Upland	Na
SW808.04	Upland	8
SW808.05	Upland	Na
SW808.06	Upland	Na
SW808.07	Upland	Na
SW808.08	Upland	Na
SW808.09	Upland	Na
SW808.10	Upland	Na
SW809.01	Upland	Na
SW809.02	Upland	Na
SW809.03	Upland	Na
SW809.04	Upland	8
SW809.05	Upland	Na
SW809.06	Upland	Na
SW809.07	Upland	Na
SW809.08	Upland	Na
SW809.09	Upland	Na
SW809.10	Upland	Na
SWG32.01	Lowland	Na
SWG32.02	Lowland	Na
SWG32.03	Lowland	Na
SWG32.04	Lowland	Na
SWG32.05	Lowland	Na
SWG32.08	Lowland	Na
SWG32.10	Lowland	4
SWG39.01	Lowland	Na
SWG39.02	Lowland	4
SWG39.03	Lowland	4
SWG39.05	Lowland	4
SWG39.06	Lowland	Na
SWG39.07	Lowland	Na
SWG39.08	Lowland	Na

SWG39.09	Lowland	Na
Timber.01	Lowland	Na
Timber.02	Lowland	Na
Timber.03	Lowland	Na
Timber.04	Lowland	Na
Timber.05	Lowland	4
Timber.06	Lowland	Na
Timber.07	Lowland	Na
Timber.10	Lowland	Na
WS4U.02	Upland	Na
WS4U.03	Upland	Na
WS4U.05	Upland	Na
WS4U.06	Upland	4
WS4U.07	Upland	Na
WS4U.08	Upland	Na
WS4U.09	Upland	Na
WS4U.10	Upland	Na
WS98-SB.01	Upland	6
WS98-SB.02	Upland	4
WS98-SB.04	Upland	4
WS98-SB.05	Upland	4
WS98-SB.06	Upland	4
WS98-SB.07	Upland	8
WS98-SB.09	Upland	8
WS98-SB.10	Upland	8

*Ploidy levels were scored using flow cytometry on one individual per population. Indications of within-population ploidy variation in the phylogenetic analysis were confirmed by subsequent flow cytometry. For methods, see [2].
