# Supplementary material for: Switchgrass Genomic Diversity, Ploidy, and Evolution: Novel Insights from a Network-Based SNP Discovery Protocol
Source: PLoS Genet. 2013 Jan 17;9(1):e1003215. doi: 10.1371/journal.pgen.1003215 (PMC3547862; doi:10.1371/journal.pgen.1003215)
Supplement: Table S3 — Quality of linkage group clustering at different call rates. (PDF) [file pgen.1003215.s017.pdf]

Table S3. Quality of linkage group clustering at different call rates.

Call rate	Number of SNPs	Number of uniquely aligned SNPs	Proportion*
0.2	178,373	10,704	60.3%
0.5	88,217	9,437	76.8%
0.9	16,700	1,809	90.8%

SNPs (0.12<MAF<0.38) at different call rates in the full-sib population were aligned to the foxtail millet genome. The uniquely aligned SNPs were clustered into the 36 linkage groups (paternal and maternal) based on the Spearman’s rank correlation coefficient of the markers.

* The proportion of uniquely aligned SNPs which were clustered into the syntenic linkage groups corresponding to a physical chromosome in foxtail millet.
