# Supplementary material for: Switchgrass Genomic Diversity, Ploidy, and Evolution: Novel Insights from a Network-Based SNP Discovery Protocol
Source: PLoS Genet. 2013 Jan 17;9(1):e1003215. doi: 10.1371/journal.pgen.1003215 (PMC3547862; doi:10.1371/journal.pgen.1003215)
Supplement: Text S1 — Technical details of UNEAK pipeline. (DOCX) [file pgen.1003215.s018.docx]

**Text S1**

**Technical details of UNEAK pipeline**

**Data storage**

In order to minimize storage space and implement efficient computation, the DNA sequences are converted to bits. Each base is stored in two bits, for example, A is 00 and C is 01. In this way, every 32 bp of sequence can be stored in a single long variable in Java. Sequences of 32, 64, 96, 128 bp, etc can be processed in UNEAK. However, to avoid the excessive sequencing errors at the end of Illumina reads, we typically use only 64 bp of each read. For example, the sequence “CTGCTTTAGCGCCTCCACCTACCTTCTTCCCC” is converted to 8790069300898029397.

**Procedures**

1. The Qseq or Fastq Files are parsed based on the barcodes. All the reads are trimmed to 64 bps and stored in multiple “tagCount” files (See data format), each of which has the reads from one sample.
2. The “tagCount” files are then compressed by collapsing identical reads into one tag, for which a count number is stored.
3. All of the “tagCount” files are merged into one master “tagCount” file, containing the tags from all of the samples.
4. The tags in the master “tagCount” file are converted to a long type array. This array is then indexed and sorted to facilitate pairwise alignment to find pairs of tags that differ by only 1 bp.
5. A homology network is constructed joining all of the tags that differ by a single base, and then a network filter is applied to find the reciprocal tag pairs (Method and Figure S1). The tag pairs are essentially SNP calls – these tag pairs are stored in a file (see data format).
6. The “tagCount” files for individual DNA samples (“taxa”) are then processed to find tags that match those in the tag pair file. In this way, a “tagsByTaxa” matrix file (see data format) is generated recording the tag distribution in all of the samples (where the “taxa” are DNA samples).
7. Based on the “tagsByTaxa” file, genotypes are called for all of the samples (“taxa”), and are output in HapMap format.

**Data format**

**TagCount**

The “tagCount” format records the tags (64 bps sequences) and their counts. For example:

Sequence Count

CTGCTTTAGCGCCTCCACCTACCTTCTTCCCCCCTCTCTCACCTACCCAATTCCAATTGGCTGA 27

CTGCTTTGCCTTGGCGTCGGATGTATCGACATTTCTCTGACAGAACTGTGCTCGCTTTTGCAT 13

**TagPair**

The “tagPair” format records the reciprocal tag pairs, which occur consecutively in the file. Each tag has a ID number. The tags with ID 0 and 1 are a pair, tags with 2 and 3 are a pair, etc. For example:

Sequence: ID

CTGCTTTAGCGCCTCCACCTACCTTCTTCCCCCCTCTCTCGCCTACCCAATTCCAATTGGCTGA 0

CTGCTTTAGCGCCTCCACCTACCTTCTTCCCCCCTCTCTCACCTACCCAATTCCAATTGGCTGA 1

CTGCTTTGCCTTGGCGTCGGATGTATCGACATTTCTCTGACAGAACTGTGCTCGCTTTTGCAT 2

CTGCTTTGCCTTGGCGTCGGATGTATCGACATTTCTCTGACAGAAGTGTGCTCGCTTTTGCAT 3

**TagsByTaxa**

The tagsByTaxa format records how many times each tag in a tag pair was observed in each of the samples (“taxa”). For example:

Tags Sample1 Sample2 Sample3

T1 3 5 7

T2 4 0 2

T3 0 1 1

T4 2 0 4

**Documentation**

More detailed documentation on usage of UNEAK can be found at <http://www.maizegenetics.net/gbs-bioinformatics>.
